# Effect of *Lactobacillus salivarius* Bacteriocin Abp118 on the Mouse and Pig Intestinal Microbiota

**DOI:** 10.1371/journal.pone.0031113

**Published:** 2012-02-17

**Authors:** Eliette Riboulet-Bisson, Mark H. J. Sturme, Ian B. Jeffery, Michelle M. O'Donnell, B. Anne Neville, Brian M. Forde, Marcus J. Claesson, Hugh Harris, Gillian E. Gardiner, Patrick G. Casey, Peadar G. Lawlor, Paul W. O'Toole, R. Paul Ross

**Affiliations:** 1 Department of Microbiology, University College Cork, Cork, Ireland; 2 School of Health Sciences, Life and Health Sciences Research Institute, University of Minho, Braga, Portugal; 3 Moorepark Food Research Centre, Teagasc, Fermoy, Ireland; 4 Department of Chemical and Life Sciences, Waterford Institute of Technology, Waterford, Ireland; 5 Alimentary Pharmabiotic Centre, University College Cork, Cork, Ireland; 6 Pig Development Department, Animal & Grassland Research & Innovation Centre, Teagasc, Fermoy, Ireland; AC Camargo Cancer Hospital, Brazil

## Abstract

Lactobacilli are Gram-positive bacteria that are a subdominant element in the human gastrointestinal microbiota, and which are commonly used in the food industry. Some lactobacilli are considered probiotic, and have been associated with health benefits. However, there is very little culture-independent information on how consumed probiotic microorganisms might affect the entire intestinal microbiota. We therefore studied the impact of the administration of *Lactobacillus salivarius* UCC118, a microorganism well characterized for its probiotic properties, on the composition of the intestinal microbiota in two model animals. UCC118 has anti-infective activity due to production of the bacteriocin Abp118, a broad-spectrum class IIb bacteriocin, which we hypothesized could impact the microbiota. Mice and pigs were administered wild-type (WT) *L. salivarius* UCC118 cells, or a mutant lacking bacteriocin production. The microbiota composition was determined by pyrosequencing of 16S rRNA gene amplicons from faeces. The data show that *L. salivarius* UCC118 administration had no significant effect on proportions of major phyla comprising the mouse microbiota, whether the strain was producing bacteriocin or not. However, *L. salivarius* UCC118 WT administration led to a significant decrease in *Spirochaetes* levels, the third major phylum in the untreated pig microbiota. In both pigs and mice, *L. salivarius* UCC118 administration had an effect on *Firmicutes* genus members. This effect was not observed when the mutant strain was administered, and was thus associated with bacteriocin production. Surprisingly, in both models, *L. salivarius* UCC118 administration and production of Abp118 had an effect on Gram-negative microorganisms, even though Abp118 is normally not active *in vitro* against this group of microorganisms. Thus *L. salivarius* UCC118 administration has a significant but subtle impact on mouse and pig microbiota, by a mechanism that seems at least partially bacteriocin-dependent.

## Introduction

Lactobacilli are Gram-positive bacteria, commonly associated with the gastrointestinal tract (GIT) of humans and animals. They are members of the Lactic Acid Bacteria (LAB) and have a number of uses in industry especially in the manufacture of dairy products [1]. Many LAB are considered to have probiotic effects. Probiotics are defined as “live microorganisms which when administered in adequate amounts confer a health benefit on the host” [2]. Several hypotheses have been proposed to explain how these probiotic microorganisms can be beneficial for the host [3]. Firstly, probiotics could enhance intestinal barrier function. Madsen and collaborators showed that the probiotic mixture VSL#3, partially comprising LAB, improved intestinal epithelial integrity and reduced its permeability, conferring protection against inflammatory luminal constituents coming from bacteria or diet [4]. They also showed that probiotic consumption conferred resistance to *Salmonella* invasion by reducing intestinal permeability [4]. Secondly, some strains demonstrate immunomodulatory activity. Indeed, in a recent study, Sierra and collaborators showed that administration of *L. salivarius* CECT5713, isolated from breast milk, improved host immunity by inducing Interleukin (IL)-10 and some immunoglobulins levels as well as inducing an increase in Natural Killer cell and monocyte numbers [5]. Furthermore, a recent study showed that *Lactobacillus salivarius* B1 can increase the number of immunocompetent cells and enhance IL-6 gene expression in the pig intestine [6]. Finally, some probiotic microorganisms exert protective effects against pathogen invasion by adhesion or metabolic competition, or inhibition due to production of antimicrobial compounds. Indeed, some *L. acidophilus* and *L. rhamnosus* strains used as probiotics can prevent enterohemorrhagic or enteropathogenic *Escherichia coli* strain adhesion to epithelial cells and thus reduce infection severity [7]. Moreover, a five-strain probiotic mixture comprising four different species (and including *Lactobacillus salivarius* DPC6005) has been shown to reduce *Salmonella* carriage in infected pigs [8].

Whereas recent studies were aimed at elucidating mechanisms by which probiotics could exert these beneficial effects, few studies have focused on if and how probiotic administration impacts the normal microbiota – either directly or indirectly. It is important to know if these microorganisms could induce alteration of the composition or activity of the host microbiota, since some microbial population alterations are associated with intestinal disorders like obesity or inflammatory bowel disease (IBD) [9], [10]. A recent study showed that administration of *Lactobacillus acidophilus* NCFM and *Bifidobacterium animalis* subsp. *lactis* Bi-07 does not affect major genera in the microbiota of children [11]. However, the specific impact of probiotic consumption on the host microbiota may depend on the identity and phenotype-specificity of the strains used. It is thus important to carry out similar studies using different known probiotic microorganisms, to increase our knowledge on the impact of probiotics on the normal microbiota, and to exclude the possibility of undesirable microbiota changes occurring.

*L. salivarius* UCC118 is well studied for its probiotic properties. Corr and collaborators have shown that this strain protects against *Listeria monocytogenes* EGDe and LO28 and *Salmonella typhimurium* UK1 infections in mice [12]. More specifically, they showed that the observed anti-*Listeria* effect was due to production of the bacteriocin Abp118 [12]. Abp118 is a broad-spectrum class IIb bacteriocin encoded by an operon located on the *L. salivarius* megaplasmid pMP118 [13]. A mutant lacking bacteriocin production still protected against S*almonella* infection whereas it did not protect against *Listeria* infection [12]. Thus, the anti-*Listeria* effect of UCC118 was due to direct antagonism via Abp118, whereas its anti-*Salmonella* effect was more likely due to competitive exclusion or immunomodulation of host defenses [12]. Because of the anti-microbial activity associated with Abp118, we hypothesized that administration of *L. salivarius* UCC118 might impact the host. The aim of this study was therefore to evaluate the impact of *L. salivarius* UCC118 administration and more specifically bacteriocin production, on the composition of the normal microbiota of healthy mice and pigs. This was addressed by determining faecal microbiota composition before and after treatment, of duration one week and four weeks in mice and pigs respectively.

## Results

### Animal model selection and construction of an isogenic *abpT* mutant in *Lactobacillus salivarius* UCC118, deficient for bacteriocin production

Previous studies have shown that two different strains of *L. salivarius*, among them UCC118, exhibited probiotic effects through anti-infective properties in mice and pigs [8], [12], [14]. Thus, these two animal models were selected for the assessment of the effect of bacteriocin production on host microbiota in these species.

In the mouse trial, animals were either sterile PBS (control group), PBS containing *L. salivarius* UCC118 expressing bacteriocin (Bac+ group) or PBS containing *L. salivarius* UCC118 deficient for bacteriocin production (Bac− group). The bacteriocin deficient mutant had been constructed in a previous study by plasmid integration into the *abpT* gene which encodes the bacteriocin transporter [12] (Table S1). To control for the possible (but unlikely) effects associated with the integrated plasmid in the genome of the knock-out strain, the strain producing bacteriocin and administered to mice in the Bac+ group was a derivative of *L. salivarius* UCC118 harboring the same plasmid but integrated in the non-essential *lacZ* gene (Table S1). Faeces of each individual of the three groups were collected at the start of the trial and after 7 days of feeding, and microbiota composition determined by amplicon pyrosequencing.

For the pig trial, all *L. salivarius* strains administered were rifampicin-resistant derivatives of the parent strains. However, the experimental facility used for the pig trial prohibited the use of antibiotic-resistant genetically-modified organisms (containing foreign DNA), and so the strains used for the mouse trial were not suitable. Thus, a new derivative of *L. salivarius* UCC118 deficient in bacteriocin production was constructed by clean deletion of the *abpT* gene. This isogenic *abpT* mutant, named *L. salivarius* UCC118 Δ*abpT*, was constructed using the pORI_19_/pVE_6007_ system previously used in our group to construct an isogenic sortase mutant in UCC118 [15] (Table S1 and Fig. S1). In this technique, 1 kb upstream and downstream flanking regions of the *abpT* gene were amplified using primers listed in Table S2, joined by Splicing by Overlap Extension (SOE)-PCR, and then cloned in the non-replicative pORI_19_ vector. This construct, named pORI-Δ*abpT* (Table S1), was introduced by transformation of *L. salivarius* UCC118 harboring the pVE_6007_ helper vector to allow replication of pORI-Δ*abpT*. Integration and then excision, leading to the deletion of the *abpT* gene, were obtained by the presence or absence of antibiotic selection pressure (Fig. S1).

Pigs were fed (each day and for 29 days) sterile milk (control group), milk containing *L. salivarius* UCC118 WT, producing bacteriocin (Bac+ group) or milk containing *L. salivarius* UCC118 Δ*abpT*, deficient in bacteriocin production (Bac− group). Pig faeces were collected at the start of the trial and after 28 days. Microbiota composition was determined by pyrosequencing of 16S rRNA gene amplicons. Fecal samples as well as ileal content and tissue samples were also collected at different times of the trial and used to enumerate administered strains in the pig GIT. Finally, pig sera were collected at the start of the trial and after 14 and 29 days of treatment, to evaluate the effect of administering the respective strains on pig immune system parameters.

### *Lactobacillus salivarius* strain UCC118 survives in the pig GIT and adheres to the ileal mucosa

**Figure 1:**
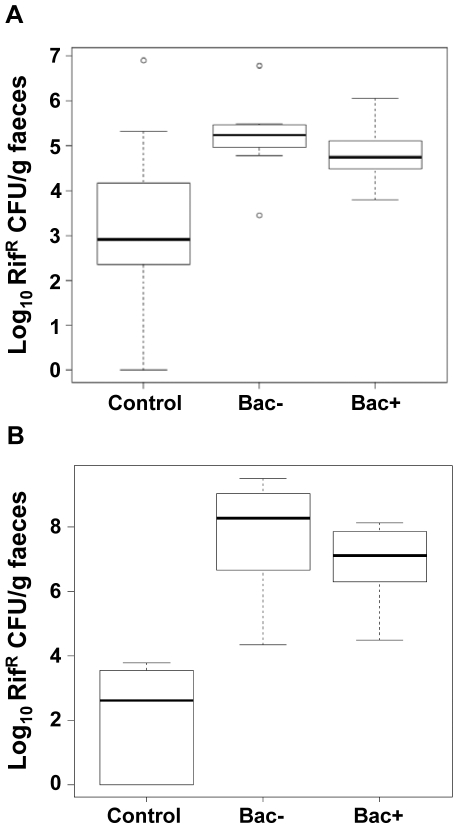
*L. salivarius* UCC118 survives transit of the pig GIT. Box-plots showing *L. salivarius* numbers on Day 14 (A) and Day 28 (B) in fecal samples collected from pigs fed sterile milk (Control group) or milk incorporating 1×10^10^ CFU/day of *L. salivarius* UCC118 WT (Bac+ group) or *L. salivarius* UCC118 Δ*abpT* (Bac− group). Box-plots represent the median and the lower and upper quartiles. Whiskers extend to the last data point still within 1.5 inter-quartile range of the quartiles.

Because of their rifampicin resistance, we were able to enumerate the administered strains in faeces throughout the pig trial and thus to determine if they were able to survive GIT transit. After 14 days treatment, all probiotic-fed pigs excreted rifampicin-resistant (Rif^R^) microorganisms between 2.8×10^3^ and 6.1×10^6^ CFU/g faeces, with a median of 5.7×10^4^ and 1.7×10^5^ CFU/g faeces for the Bac+ group and the Bac− group, respectively (Fig. 1A). However, a median of 8.0×10^2^ Rif^R^ CFU/g faeces was enumerated in control pig faeces and statistical analysis revealed that control group counts were not significantly different from counts in Bac+ or Bac− groups after multiple testing (*p* = 0.058 and 0.046, respectively; Bonferroni Correction>0.1) (Fig. 1A). This was due to presence of abundant naturally Rif^R^ microorganisms in two of the control group pigs which excreted 8×10^6^ and 2×10^5^ Rif^R^ CFU/g faeces, respectively, whereas the other pigs excreted between 0 and 1×10^3^ Rif^R^ CFU/g faeces (Fig. 1A).

Following 28 days treatment, Bac+ and Bac− groups excreted Rif^R^ microorganisms at median levels of 1.4×10^7^ and 3.0×10^8^ CFU/g faeces, respectively (Fig. 1B). This was significantly higher than similar counts in the control group (9.0×10^2^ Rif^R^ CFU/g faeces, *p*<0.001, Bonferroni Correction<0.01) (Fig. 1B). This shows that *L. salivarius* UCC118 WT or Δ*abpT* were the major microorganisms responsible for these counts. The presence of both strains in pig faeces demonstrates that *L. salivarius* UCC118 survived transit of the pig GIT. Both strains seemed to survive similarly as there was no significant difference between *L. salivarius* UCC118 fecal numbers in the two probiotic-fed groups (Fig. 1B).

**Figure 2:**
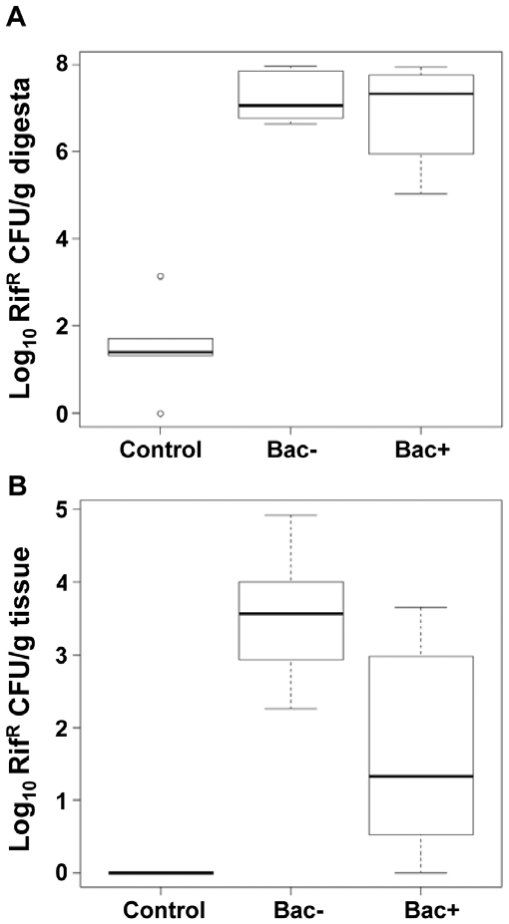
*L. salivarius* UCC118 survives in, and adheres to, the pig ileum. Box-plots showing *L. salivarius* numbers on Day 29 in ileal digesta (A) and tissue (B) samples collected from pigs fed sterile milk (Control group) or milk incorporating 1×10^10^ CFU/day of *L. salivarius* UCC118 WT (Bac+ group) or *L. salivarius* UCC118 Δ*abpT* (Bac− group). Box-plots represent the median and the lower and upper quartiles. Whiskers extend to the last data point still within 1.5 inter-quartile range of the quartiles.

Enumeration of Rif^R^ microorganisms was also performed with ileum tissue and its content after 29 days of treatment. Rif^R^ microorganisms were found in the ileal contents of every pig from the probiotic-fed groups. All pigs from the Bac+ group, but only 6 of the 8 pigs from the Bac− group, harbored Rif^R^ microorganisms on the ileal mucosa. A median of 2.7×10^7^ and 1.3×10^7^ Rif^R^ CFU/g digesta were enumerated in ileal contents from pigs of the Bac+ and Bac− groups, respectively (Fig. 2A). These counts were significantly different from control group counts (2.5×10^1^ Rif^R^ CFU/g digesta) and thus associated to *L. salivarius* UCC118 WT or Δ*abpT* presence in ileal digesta (*p*<0.005 and Bonferroni Correction<0.05) (Fig. 2A). Similarly, a median of 2.5×10^1^ and 4.9×10^3^ Rif^R^ CFU/g tissue were enumerated on ileal tissues from pigs of the Bac+ and Bac− groups respectively (Fig. 2B). Again, these counts were significantly different from control group counts (no Rif^R^ CFU for any of the control group pigs) and thus associated to *L. salivarius* UCC118 WT or Δ*abpT* presence (*p*<0.005 and Bonferroni Correction<0.05) (Fig. 2B). This allowed us to demonstrate that administered strains were able to survive in, and colonize upon, the ileal mucosa. We also observed that the mutant strain seemed to colonize slightly better than the wild-type strain but this difference failed to be significant after multiple testing (*p*<0.05 and Bonferroni Correction>0.1) (Fig. 2B).

### *Lactobacillus salivarius* UCC118 administration affects average daily gain and feed conversion efficiency parameters in pigs for a small temporal window only

Pigs were individually housed and individual pig weights were recorded on Day 0. Pig weight and feed removal were recorded after 14 and 28 days of treatment to allow calculation of average daily gain, average daily feed intake and feed conversion efficiency. At the start of the trial, all three groups had similar pig weight (*p*>0.10) (Table 1). Similarly, pig weight was similar for all three treatments at Day 14 (*p*>0.10) and Day 28 (*p*>0.10) of the trial (Table 1). In addition, average daily feed intake of all three groups was similar at Day 14 (*p*>0.10) and Day 28 (*p*>0.10) (Table 1). However, between Day 14 and Day 28 of the trial, average daily gain was higher for control pigs than for probiotic-fed pigs (*p* = 0.006) (Table 1). The lower average daily gain observed in the probiotic-fed group was associated with a numerical deterioration in feed conversion efficiency (*p* = 0.06) compared to the control, during this period. This difference in average daily gain was not observed between Day 0 and Day 14 (*p*>0.10) or between Day 0 and Day 28 (*p*>0.10) (Table 1). However, feed conversion efficiency also tended to be poorer (*p* = 0.09) for probiotic-fed pigs than the control pigs between Day 0 and Day 28 (Table 1).

**Table 1:** *L. salivarius* UCC118 administration effect on pig growth performance.

	Control(a)	Bac+(a)	Bac−(a)
**Weight (kg)**			
Day 0	12.7±0.4	12.7±0.5	12.8±0.4
Day 14	22.2±0.7	22.3±0.8	22.6±0.7
Day 28	35.1±0.9	33.6±0.8	34.4±0.7
**Average daily gain (g)**			
Day 0–14	675±35.5	688±27.6	698±29.1
Day 14–28	920±24.4	848±17.9*	807±24.4*
Day 0–28	798±25.8	748±18.2	774±16.6
**Average daily feed intake (g)**			
Day 0–14	747±36.9	753±28.3	775±34.0
Day 14–28	1199±28.9	1171±45.9	1214±32.8
Day 0–28	973±30.1	962±35.0	995±27.1
**Feed conversion efficiency**			
Day 0–14	1.11±0.012	1.10±0.013	1.11±0.015
Day 14–28	1.31±0.029	1.45±0.053++	1.44±0.048++
Day 0–28	1.22±0.018	1.29±0.029+	1.29±0.024+

(a) Values are mean ± SEM, *n* = 8.

*p = 0.006,

++ p = 0.06 and

+ p = 0.09, between the Bac+ and Bac− groups and the control group.

We also attempted to measure IL-10 and IL-8 levels in the treated animals; IL-10 could not be detected by the assay employed, and there were no statistically significant differences between IL-8 levels in treatment groups (data not shown).

### Mouse and pig microbiota evolved during the time of the trial

Total DNA was extracted from murine and porcine faeces at the beginning of the trial and after 7 and 28 days of treatment respectively, and used to amplify and sequence pooled amplicons of the V4 (mouse trial) or V4–V5 (pig trial) region of the bacterial 16S rRNA gene. Each sequence read corresponded to a specific operational taxonomic unit (OTU) and was assigned at the phylum and genus level by homology comparison. The number of reads per OTU allowed us to determine the relative abundance of each OTU.

**Figure 3:**
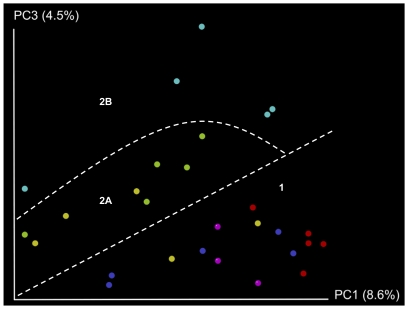
Separation of mouse microbiota by treatment group and time. King plot showing unweighted UniFrac-based PCoA. Each dot represents an individual at either the start of the trial (Day 0) or after seven days of treatment (Day 7). Day 0: control group (dark blue), Bac+ group (red), and Bac− group (purple); Day 7: control group (green), Bac+ group (light blue), and Bac− group (yellow).

**Figure 4:**
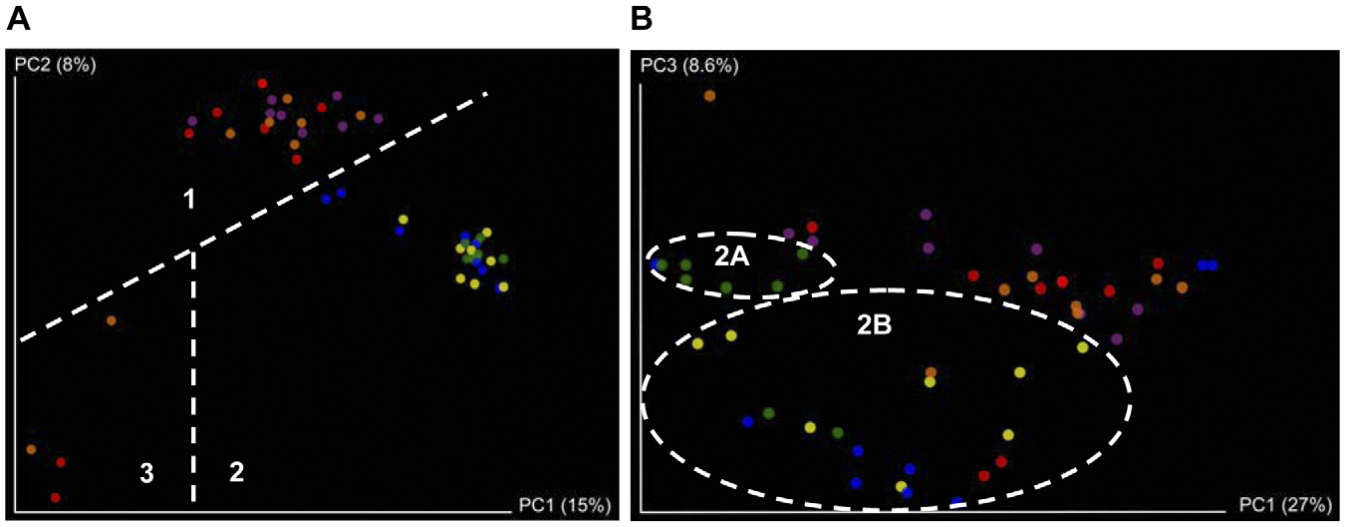
Separation of porcine microbiota by treatment group and time. King plots showing PCoAs based on (A) unweighted UniFrac data and (B) weighted UniFrac data. Each dot represents an individual at either the start of the trial (Day 0) or after 28 days of treatment (Day 28). Day 0: control group (orange), UCC118 Bac+ group (purple), UCC118 Bac− group (red) and Day 28: control group (green), UCC118 Bac+ group (yellow), UCC118 Bac− group (blue).

Principal Coordinates Analysis (PCoA) was used to highlight similarities or differences between each individual animal on the basis of their microbiota composition and of the time of sampling (Day 0 and 7 for the mouse trial and Day 0 and 28 for the pig trial). This analysis revealed that mice, on Day 0, could not be discriminated on the basis of microbiota composition as they all clustered together (cluster 1 = Day 0, Fig. 3). It also allowed us to show that most individual pigs (19 of 24 pigs) could not be discriminated on the basis of their microbiota composition determined on the first day of the trial (cluster 1 = Day 0, Fig. 4A). However 5 pigs (3 from the control group and 2 from the Bac− group) demonstrated a microbiota that was dramatically different from that of cluster 1 pigs because their microbiota seemed to be depleted in a number of genera (cluster 3, Fig. 4A). The two pigs from the Bac− group had diarrhea on the first day of the trial. We thus conclude that their abnormal microbiota composition reflects this condition. The three other pigs from the control group also demonstrated an abnormal microbiota composition that was similar to the microbiota of the two pigs with diarrhea, although these animals did not show any physical symptoms of disease. Therefore, data from the five pigs with an abnormal microbiota composition were excluded from further Day 0 data analysis.

**Figure 5:**
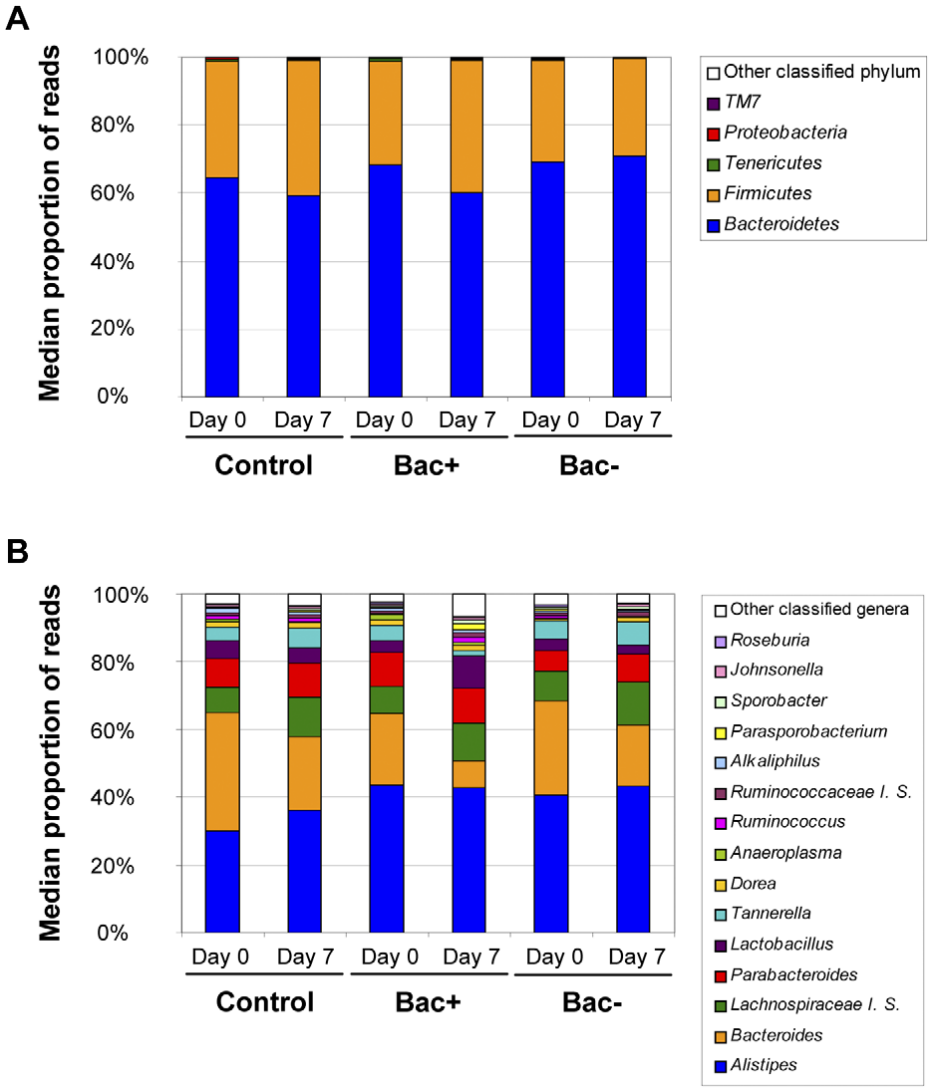
Microbiota composition in mice. Graphs represent the microbiota composition at the phylum (A) or genus (B) level in mice at the start of the trial (Day 0) and after 7 days of treatment (Day 7). Values represent the median proportions of classified reads for each phylum or genus as determined by the RDP classifier and for each treatment group: Control Bac+ and Bac−.

Determination of microbiota composition at the start of the trial revealed that the mouse microbiota was composed of a majority of *Bacteroidetes* and *Firmicutes*, accounting for almost 95% of total microbiota (Fig. 5, Table S3). In the pig, these two phyla were also a majority and represented around 85% of total microbiota. Comparison of OTU relative abundance between each group (control, Bac+ and Bac−) confirmed PCoA in showing that there was no significant difference between the initial microbiota composition of each group, neither at the phylum nor at the genus level (data not shown). This is significant because it would allow us to identify any probiotic-administration related effect on microbiota composition.

**Figure 6:**
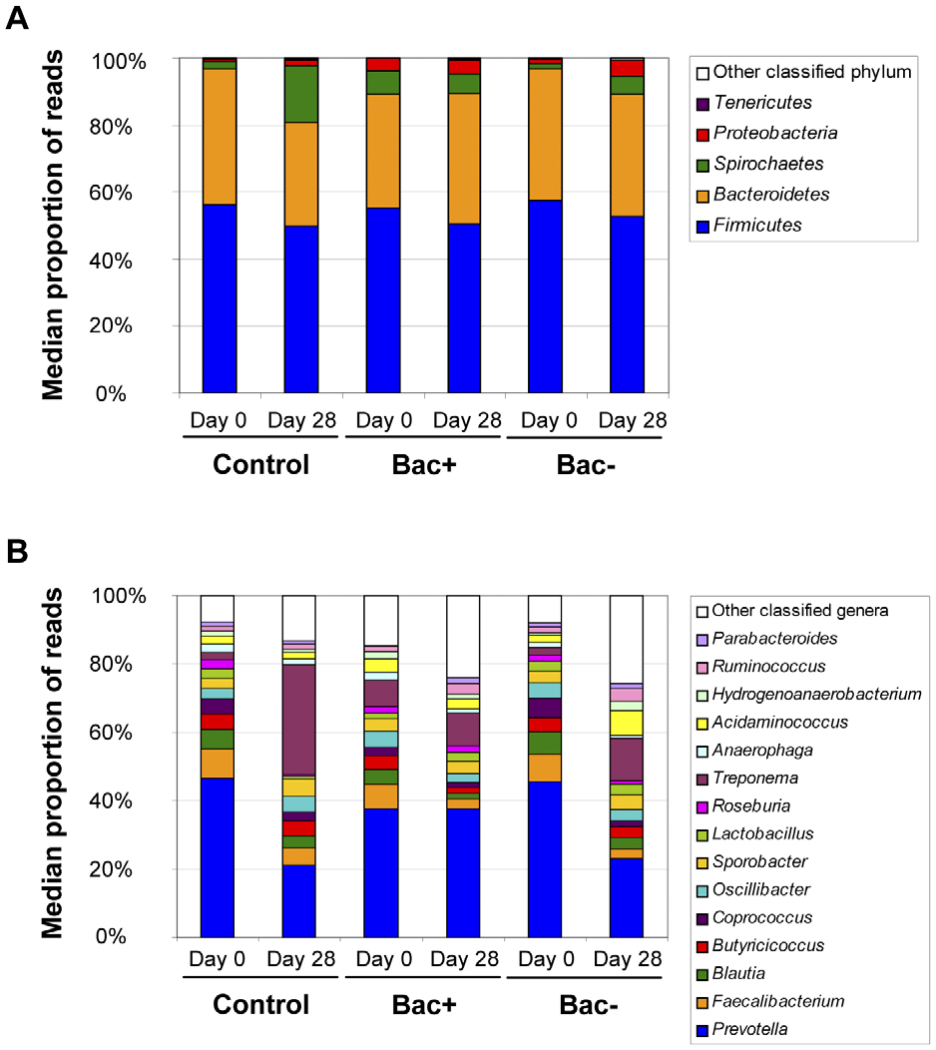
Microbiota composition in pigs. Graphs represent the microbiota composition at the phylum (A) or genus (B) level in pigs at the start of the trial (Day 0) and after 28 days of treatment (Day 28). Values represent the median proportions of classified reads for each phylum or genus as determined by the RDP classifier and for each treatment group: Control Bac+ and Bac−.

PCoA also revealed that animal microbiota composition determined at the end of each trial (after 7 days of treatment for the mouse trial and 28 days for the pig trial) seemed to differ from the microbiota determined at Day 0 (Cluster 2 = Day 7 and Day 28, Fig. 3 and 4 respectively) with the exception of two mice from the Bac− group that tended to cluster with Day 0 mice, on the basis of their microbiota composition. To evaluate microbiota evolution over time, and bearing in mind that we had established that animal microbiota composition was similar at the start of the trial, we compared relative abundance of all OTUs at the start and the end of the trial in the control group only. We first observed in the murine microbiota, small and non significant variations over time of a number of genera (Table S3). In contrast, one major pig microbiota phylum significantly increased in proportion during pig growth. In fact, *Spirochaetes* proportion represented a median of 0.02 of the microbiota at the start of the trial whereas it represented a median of 0.15 at the end (p<0.005, Bonferroni correction<0.05) (Fig. 6A, Table S4). Probably in compensation, *Firmicutes* and *Bacteroidetes* proportions tended to decrease slightly, but not significantly (Fig. 6A and Table S4). The same variation was observed at the genus level whereby the proportion of *Treponema*, a member of the phylum *Spirochaetes*, significantly increased during the time of the trial (median of 0.01 in proportion at Day 0 compared to a median of 0.15 at Day 28, *p*<0.005 and *q*<0.05) while members of the phylum *Firmicutes* such as *Blautia*, *Clostridium*, *Faecalibacterium*, *Roseburia* or *Parasporobacterium* significantly decreased in proportion (Fig. 6B and Table S4). In addition, proportions of *Butyricimonas* and *Galbibacter* (CFB group bacteria), *Orientia*, *Sutterella*, *Desulfovibrio* and *Actinobacillus* (*Proteobacteria*) and *Methanosphaera* (*Euryarchaeotes*) also changed significantly during the time course of the trial (Table S4). All these modifications presumably represent the normal development of the microbiota during pig or mouse maturation.

### *Lactobacillus salivarius* UCC118 administration induces modification of the mouse microbiota by a bacteriocin-dependent mechanism

PCoA revealed a clear separation, at the end of the mouse trial, between the control and Bac− groups, and the Bac+ group (cluster 2A: control and Bac− groups, Day 7 and cluster 2B: Bac+ group, Day 7; Fig. 3). Comparisons of OTU relative abundance between each group allowed identification of specific taxa responsible for this differentiation. In fact, we observed that proportion of *Bacteroidetes* decreased whereas *Firmicutes* increased more in Bac+ treated mice compared to control and Bac− treated mice (Fig. 5A and Table S3). However, changes observed at the phylum level were not statistically significant after correction for multiple testing. Similarly, there was no significant difference at the genus level between microbiota composition of mice of the control group or of the Bac− group when compared to all the other groups. Interestingly, a number of genus proportions were significantly altered by *L. salivarius* UCC118 administration when comparing the microbiota composition of Bac+ group mice to that of control and Bac− group mice (Fig. 5B and Table S3). Thus, we observed a greater decrease in the proportion of *Bacteroides* (*Bacteroidetes*), one of the largest genera in the mouse microbiota, in Bac+ mice than in other mice (*p*<0.001, *q*<0.05) (Fig. 5B and Table S3). Proportions of two other members of the *Bacteroidetes* phylum were affected by *L. salivarius* UCC118 administration. *Tannerella* decreased in Bac+ mice whereas it increased in other groups and *Prevotella* showed a greater increase in Bac+ mice than in other mice (*p*<0.005, *q*<0.05 and *p*<0.0005, *q*<0.05, respectively) (Fig. 5B and Table S3). Proportions of four members of the *Firmicutes* phylum, *Parasporobacterium*, *Faecalibacterium*, *Papillibacter* and *Ethanoligenens* were significantly higher in mice from the Bac+ group than in mice from other groups (*p*<0.005, *q*<0.05; *p*<0.01, *q*<0.05; *p*<0.01, *q*<0.05 and *p*<0.005, *q*<0.05, respectively) (Fig. 5B and Table S3). We conclude from these results that *L. salivarius* UCC118 administration affected genera in the *Bacteroidetes* and *Firmicutes* phyla. As these effects were only observed when the wild-type strain was administered, we can say that bacteriocin production was mainly responsible for *L. salivarius* UCC118 administration effects on mouse microbiota.

### *Lactobacillus salivarius* UCC118 administration induced modification of the pig microbiota which was greater with a bacteriocin producing strain

PCoA analysis showed that the microbiota composition of probiotic-fed pigs tended to cluster together and tended to differ from control pigs (cluster 2A: control group, Day 28 and cluster 2B: Bac+ and Bac− groups, Day 28; Fig. 4B). One pig from the Bac− group, at Day 28, did not cluster with any of the other pigs for yet unknown reasons and was considered as an outlier; data from this pig were excluded from further analyses. Comparison of OTU relative abundance between each group allowed identification of specific taxa responsible for differentiation between groups. Thus, we observed that the proportion of *Spirochaetes*, one of the largest phyla in the pig microbiota, was measurably affected by *L. salivarius* UCC118 administration. Whereas, as already stated, *Spirochaetes* proportion significantly increased over time in control pig microbiota, its proportion remained constant in Bac+ pig microbiota (Fig. 6A and Table S4). Thus, the proportion of *Spirochaetes* was significantly lower in Bac+ pig microbiota than in control pig microbiota after 28 days treatment (*p*<0.005, Bonferroni correction<0.05) (Fig. 6A and Table S4). In the Bac− group, we observed an intermediate state: the proportion of *Spirochaetes* tended to increase but to a lesser extent to what occurred in the control group (Fig. 6A and Table S4). After statistical analysis, *Spirochaetes* proportion in the Bac− pig microbiota was significantly lower compared to that in control pig microbiota when only considering *p* value, but it did not reach significance after adjustment for multiple testing (*p*<0.05, Bonferroni correction>0.5). Moreover, *Spirochaetes* proportion in Bac− pig microbiota was not significantly different from the proportion observed in Bac+ pig microbiota. We observed the same phenomenon at the genus level, where the trend for *Treponema* (*Spirochaetes*) evolved in the same way as the whole phylum did (Fig. 6B and Table S4). *Treponema* proportion was thus significantly lower in microbiota of the Bac+ pigs than in the control pigs (*p*<0.005, *q*<0.05). The *Treponema* proportion of the Bac− pig microbiota was lower compared to the proportion of *Treponema* in control pigs (*p*<0.05) but this difference was not significant after multiple testing (*q*>0.1). Moreover, *Treponema* proportion in Bac− treated pig microbiota was not significantly different from proportion observed in the Bac+ group. A number of *Firmicutes* genera were also affected by *L. salivarius* UCC118 administration during the pig trial. Thus, we observed that whereas the proportion of *Sudboligranulum* increased in all three groups during the trial, a significantly greater increase occurred in the Bac+ group compared to the control group (*p*<0.005, *q*<0.05) (Table S4). While the proportion of *Oribacterium* decreased in the control group over time, it increased in both probiotic-fed pig groups, but this difference was only significant when comparing Bac+ and control groups (*p*<0.005, *q*<0.05) (Table S4). *Anaerostipes* proportion tended to slightly increase over time in the control group whereas it decreased in both the Bac+ and Bac− groups. Thus, proportion of *Anaerostipes* was significantly lower in the Bac+ group than in the control group (*p*<0.005, *q*<0.05). Proportion of *Anaerostipes* in the Bac− group was not statistically different from levels observed in the two other groups (Table S4). *Lactonifactor* proportion decreased with time in the three groups but the decrease was significantly higher in Bac+ group (*p*<0.005, *q*<0.05) (Table S4). Finally, the proportion of *Hallella* (*Bacteroidetes*) decreased in the control group whereas it tended to remain constant over the time in both Bac+ and Bac− groups (Table S4). The *Hallella* proportion was thus significantly higher in the Bac+ group compared to the control group (*p*<0.01, *q*<0.05) whereas it was not different from *Hallella* proportion in the Bac− group. We can conclude from these results that administration of *L. salivarius* UCC118 WT significantly affected pig microbiota leading to significant differences between microbiota composition of control and Bac+ groups. We observed that administration of the mutant strain, *L. salivarius* UCC118 Δ*abpT*, led to an intermediate state: the porcine fecal microbiota composition in this group was not statistically different from either of the other two groups, but tended to be more similar to the microbiota recorded for the Bac+ group. This result concurs with PCoA which showed that Bac+ and Bac− pigs tend to cluster together and tend to differ from control pigs (Fig. 4B). We can thus say that bacteriocin production may be only partially responsible for the *L. salivarius* UCC118 administration effects on the pig microbiota. Other mechanisms could be involved and need to be identified.

### Administration of probiotic *L. salivarius* UCC118 did not significantly alter intestinal microbiota diversity in mice or pigs

To measure global effects upon microbiota composition wrought by *L. salivarius* UCC118 administration, we calculated the Shannon Index and Phylogenetic Diversity for the control and treatment groups pre- and post-administration (Figure S2). In the mouse trial, the Phylogenetic Diversity, but not the Shannon Index, increased over the 7 days of the intervention. There was no significant difference within or between any of the groups at either time point (Fig. S2A; Results not shown for between-group analysis). The bar chart of the data gives the impression that some of the comparisons, particularly within the Bac− and Bac+, would have significant p-values, but the small sample sizes and large variation within the samples lead to non-significance.

In the pig trial, the alpha diversity in the control group at four weeks was significantly different from alpha diversity at one week for both Shannon diversity and Phylogenetic Diversity (Fig. S2B). The Bac− group showed a significant difference in Phylogenetic Diversity only, but neither measure of alpha diversity was significantly different between Day 0 and Day 28 in the Bac+ group (Fig. S2B). There were no significant differences between any of the groups at either time point (Results not shown).

Because of slightly longer average read lengths, we were able to identify some dominant lactobacilli in the murine faecal samples, but not the porcine samples. The predominant *Lactobacillus* species (among those identifiable) were *L. aviarius*, *L. equicursoris*, *L. intestinalis*, *L. salivarius*, and *L._vitulinus*, with *L. intestinalis* being numerically most abundant. However, the Kruskal-Wallis test showed a *p*-value of 0.21 (Kruskal-Wallis chi-squared = 7.13, df = 5), which shows that there is no difference in the number of *L. intestinalis* between any pair of groups in the dataset.

## Discussion

The aim of this study was to assess the effect of the administration of *L. salivarius* UCC118 and more specifically the effect of bacteriocin production by this microorganism on the intestinal microbiota of mammalian models, using faeces as a surrogate. For simplicity, we determined microbiota composition pre- and post-intervention periods of one and four weeks in mice and pigs, respectively. Shorter duration microbiota perturbations may have been overlooked by this pragmatic trial design. Overall, two measures of microbiota diversity, Shannon index and Phylogenetic Diversity, did not alter significantly within or between treatment groups, but as noted above, large inter-individual variation within treatment groups may confound this measurement.

Our observation that *L. salivarius* UCC118 survived pig gastrointestinal tract and colonized the pig ileum, is concordant with the behavior of another *L. salivarius* strain, DPC6005 [14], [16]. Moreover, Walsh and collaborators showed that among a five strain probiotic mixture, *L. salivarius* DPC6005 was predominantly recovered from ileal digesta and mucosa compared to the 4 other *Lactobacillus* species used in the mixture [14]. The authors hypothesized that this was due to a competitive advantage afforded by Salivaricin P produced by *L. salivarius* DPC6005 [14]. The bacteriocin Abp118 produced by *L. salivarius* UCC118, is highly similar to Salivaricin P [17] and we thus hypothesized that its production would also confer an advantage to *L. salivarius* UCC118 intestinal colonization. However, our data do not provide a straightforward confirmation of this hypothesis. Indeed, we observed no significant difference between the colonizing ability of *L. salivarius* UCC118 WT and its non-bacteriocin-producing derivative in pigs. On the contrary, *L. salivarius* UCC118 Δ*abpT* tended to be present in higher numbers on porcine ileal mucosa than the wild-type strain. It thus seems that under the conditions employed, *L. salivarius* UCC118 ileal colonization was not due to bacteriocin production. It should be noted that in our study, *L. salivarius* UCC118 was administered alone and not as a member of a probiotic mixture. It thus did not need to compete with other members of the inoculum as in the study of Walsh and collaborators [14]. So, it can be hypothesized that the apparent predominance of *L. salivarius* DPC6005 in the porcine ileal mucosa was not due to competition with member(s) of the host microbiota, but was due to competition with the other components of the probiotic mixture. However, as molecular methods could not be applied in this study, we assed the number of administered probiotics by bacterial cell enumeration. This technique may not be the most accurate but gives a good overview of the capacity of the probiotic strain to survive and colonize within the GIT.

We also showed in this study that between Day 14 and Day 28 of the trial, pigs fed probiotics gained significantly less weight than pigs in the control group. This was associated with higher feed conversion efficiency during this period, for these groups compared to the control group. This result contradicts the finding of a previous study that found that administration of *Lactobacillus reuteri* BSA131 to piglets enhanced pig daily weight gain and feed conversion rate [18]. However, our results are in accordance with a recent study showing that intake of *Lactobacillus gasseri* SBT2055 by adults with obese tendencies significantly reduced body weight and body fat mass among other parameters [19]. We can conclude that the probiotic effect on body weight is species and strain dependent. While body weight enhancement can be useful in animal production, the opposite effect would be very useful for human weight management. Further studies including measurement of probiotic effect on other host factors like adiponectin or calprotectin are needed to confirm this possibility.

Many lactobacilli have been shown to modulate host immunity [5], [6], [14]. We attempted to measure porcine IL-10 levels but despite several attempts to improve the protocol, we were not able to detect IL-10 in pig serum. *L. salivarius* UCC118 consumption had no effect on IL-8 levels in pig. We cannot exclude the possibility that production of other cytokines such as IL-12 or IL-6 might be modulated by UCC118, as shown for other *L. salivarius* strains [6], [20].

Microbiota composition of control animals revealed that the major phyla in pigs were (in decreasing order of importance): *Firmicutes*, *Bacteroidetes*, *Spirochaetes* and *Proteobacteria*. These results are in accordance with a previous study carried on by Lamendella and collaborators [21] where they showed that the most abundant phyla in pigs are *Firmicutes* and then *Bacteroidetes*. According to this study, two other phyla are also present in great amount in pigs: *Proteobacteria* and *Spirochaetes*. Similarly, in the mouse trial, we determined that the major microbiota phyla were (in decreasing order of importance): *Bacteroidetes*, *Firmicutes*, *Tenericute*, *Proteobacteria* and *TM7*. These results are in accordance with results of a previous study where the authors showed that the relative abundances of the major phyla were: *Firmicutes* 30–70%; *Bacteroidetes*, 10–40%; *Proteobacteria* 1–15%; *Actinobacteria*, *Tenericutes*, *TM7*, and *Verrucomicrobia* 0.1–0.5% [22].

The design of these studies allowed us to determine what microbiota alterations were due to probiotic administration, or to bacteriocin production. Despite administration of a large inoculum of *L. salivarius* UCC118 to animals (around 1×10^10^ CFU/day), total proportions of *Lactobacillus* in microbiota of probiotic fed animals did not change over the time course of the trials. We can hypothesize that *L. salivarius* UCC118 replaced other members of the *Lactobacillus* genus. In mice and pigs, members of the phylum *Firmicutes* were affected either positively or negatively by *L. salivarius* UCC118 consumption. In both trials, this effect was observed when the wild-type cells were administered, allowing us to conclude that this effect is bacteriocin dependent. Reduction in proportion of certain *Firmicutes* genera was foreseen, as Abp118 is active on closely related microorganisms i.e. *Firmicutes* members [23]. If some bacteriocin-sensitive *Firmicutes* members decrease in proportion, others will have the opportunity to replace them, explaining our observation that some *Firmicutes* members increased in proportion. Elimination-succession could be a general phenomenon related to bacteriocin action upon microbiota composition.

Probiotic consumption also affected Gram negative microorganisms in both mouse and pig models. This is a surprising and interesting result because, as stated above, Abp118 has been shown *in vitro* to be active only against closely related species, and bacteriocins elaborated by LAB are normally active exclusively against Gram positive microorganisms [23], [24], [25]. Moreover, Corr and collaborators showed that Abp118 is not active against *Salmonella typhimurium*, and hypothesized that the *L. salivarius* UCC118 protective effect against this pathogen is more likely due to competitive exclusion or immunomodulation of host defenses [12]. However, LAB bacteriocin activity against Gram negative microorganisms can be detected under certain *in vitro* conditions e.g. when the outer membrane is permeabilized as when EDTA treated [24], [26]. Moreover, a novel bacteriocin produced by *L. salivarius* 1077 was identified in a recent study and demonstrated activity against a number of Gram negative genera such as *Salmonella* and *Campylobacter*, both *in vitro* and *in vivo* [27]. The reductions we observed in proportions of *Bacteroides* and *Tanerella* (*Bacteroidetes*) in mice administered *L. salivarius* UCC118 WT when compared to the control, might exemplify anti-Gram negative bacterium activity of LAB bacteriocins under *in vivo* conditions. We also showed that *Treponema* (*Spirochaetes*) proportions were significantly lower in pigs fed *L. salivarius* UCC118 WT, when compared to the control. However, *Treponema* levels also tended to be lower in pigs fed bacteriocin deficient *L. salivarius* UCC118 cells, demonstrating that bacteriocin production may not be entirely responsible for the observed effect. In this context, it is interesting to note that a recent metagenomic study identified unusually high levels of certain *Spirochaetes* in the porcine gut, relative to other mammals [21]. Thus the ability of *L. salivarius* to modulate these levels could be significant. In addition to bacteriocin production, other yet-unknown bacterial properties such as competition for intestinal niches (competitive exclusion), may also be involved in *Spirochaetes* inhibition by *L. salivarius*. It should be noted that the Gram negative genera *Prevotella*, in mouse, and *Hallella*, in pig, showed greater proportions in the Bac+ treated group compared to the control group. This effect can be explained, as for *Firmicutes*, by the fact that if some genera tend to decrease, other can thus replace the former and expand in proportion. The biological significance of alterations caused upon levels of gram-negative bacteria is currently unclear, but could be significant. For example, *Prevotella* can cause opportunistic infection in humans and some *Tannerella* strains can act as anaerobic periodontal pathogens [28], [29]. As noted above, Gram-negative bacteria are typically insensitive *in vitro* to bacteriocins from gram positive bacteria because of exclusion by the outer membrane; this exclusion may be less effective *in vivo*, for example by chelation of divalent cations that stabilize this outer membrane. Further research is required to determine precisely how *L. salivarius* UCC118 can induce inhibition of certain Gram negative microorganisms.

*L. salivarius* UCC118 modulation of Gram negative bacterium proportions is interesting because it supports the concept of a counter-pathogen effect by a probiotic. *Bacteroidetes* and *Spirochaetes* include natural members of the intestinal microbiota that, under certain conditions, can become opportunistic pathogens in both humans and animals. *Treponema* sp. has been shown to induce colitis, and *Bacteroides* sp. synthesizes a toxin that can lead to diarrhea in the infected host [30], [31]. Administration of cultures like *L. salivarius* UCC118 could afford animals some degree of protection from these kinds of infections. It is also noteworthy that microbiota alterations linked to *L. salivarius* UCC118 administration were limited to a small number of genera and are clearly less dramatic than microbiota disruption caused by antibiotic treatment [32], [33]. The fact that *L. salivarius* UCC118 did not cause dramatic alteration in the fecal microbiota is also reassuring, as the consumption of probiotic products should not lead to potentially drastic microbiota re-modeling. Such a conclusion cannot be generalized, however, and will be qualified in future studies by reference to strain identity, by dosage, and by sampling the microbiota throughout the GIT length, rather than just in faeces.

## Materials and Methods

### Ethics Statement

Experiments with mice were approved by the UCC Animal Experimentation Ethics Committee and experimental procedures were conducted under license from the Irish Government (license number B100/3729).

The pig experiments described below complied with European Union Council Directive 91/630/EEC (outlines minimum standards for the protection of pigs) and European Union Council Directives 98/58/EC (concerns the protection of animals kept for farming purposes) and was approved by, and a license obtained from, the Irish Department of Health and Children (license number B100/4147).

### Bacterial strains and culture conditions

The bacterial strains and plasmids used in this study are listed in Table S1. *Lactobacillus salivarius* strain UCC118 and its derivatives were grown at 37°C under microaerophilic conditions (5% CO_2_) in de Man-Rogosa-Sharpe (MRS) medium [34] (Oxoid Ltd., Basingstoke, Hampshire, United Kingdom). When required, erythromycin (4 µg/ml), chloramphenicol (4 µg/ml), or rifampicin (150 µg/ml) were added to the culture media. *Escherichia coli* EC101 and *Lactococcus lactis* LL108 were used in this study as plasmid hosts. *E. coli* EC101 was cultured under vigorous shaking at 37°C in LB medium [35] (Oxoid Ltd., Basingstoke, Hampshire, United Kingdom) with kanamycin (25 µg/ml) and erythromycin (300 µg/ml) when required. *L. lactis* LL108 was cultured without shaking at 30°C in M17 medium containing 0.5% glucose [36] (Oxoid Ltd., Basingstoke, Hampshire, United Kingdom) with erythromycin (5 µg/ml) when required.

### DNA manipulations

The primers used in this study were purchased from MWG Biotech (Ebersberg, Germany) and are listed in Table S2. For cloning purposes, Pwo DNA Polymerase (Roche, Mannheim, Germany) was used for PCR amplification, while for screening purposes BIOTAQ DNA Polymerase (Bioline, London, United Kingdom) was used. Restriction endonucleases, T4 DNA ligase, and PCR purification kits were purchased from Roche and used according to the manufacturer's recommendations. *E. coli* EC101 and *L. lactis* LL108 were used as hosts for pORI_19_ constructs. Plasmid DNA was isolated from *E. coli* and *L. lactis* by using the QIAprep spin Miniprep kit (QIAGEN, Crawley, United Kingdom) adapted for use with lactococci by the incorporation of 20 mg/ml lysozyme (Sigma, St. Louis, MO) in the first buffer. Genomic DNA of *L. salivarius* UCC118 was isolated as previously described [23]. *L. salivarius* UCC118 was transformed by using a procedure described by van Pijkeren et al. [15].

### Creation of an *abpT* deletion mutant

The different steps involved in the creation of the mutant and the verification of the clean deletion are presented in Figure S1. Genomic DNA of *L. salivarius* UCC118 was used as a template for PCR amplification of the 5′- and 3′-end-flanking regions of the *abpT* gene (LSL_1910), using primer pairs *abpT-US for*/*abpT-US rev* and *abpT-DS for*/*abpT-DS rev* (Table S2). The amplicons were joined by SOE-PCR using the primer pair *abpT-US for*/*abpT-DS rev* (Table S2). The resultant 2 kb amplicon was digested using *Bam*HI and *Eco*RI and cloned into pORI_19_ digested with the same enzymes. The resultant plasmid was named pORI-Δ*abpT* (Table S1 and Figure S1A). The *abpT* clean deletion mutant in *L. salivarius* UCC118 was constructed as described previously [15]. Deletion of the *abpT* gene was further confirmed by PCR amplification using the primer pair *abpT KO for*/*abpT KO rev*, which flanks the *abpT* gene (Table S2) and by Southern hybridization (Fig. S1B). Absence of bacteriocin production was confirmed as previously described [12] (Fig. S1C).

### Mouse feeding trial

15 Female Balb/c mice aged 8–10 weeks were used for the mouse trial. During the treatment period, in addition to a standard rodent diet, each mice in the probiotic groups were administered 1×10^10^ CFU of *L. salivarius* UCC118 *lacZ* (Bac+ group) or *L. salivarius* UCC118 *abpT* (Bac− group) (Table S1) in 0.1 ml PBS by oral gavage daily for 7 days as was done by Bernbom and collaborators [37]. A control group of mice was fed sterile PBS (Gibco, Paisley, Scotland) daily for 7 days, in addition to a standard rodent diet. At all times during the trial, mice were provided with *ad libitum* access to fresh water. Five mice were used per group. Administered cultures were prepared as follows: every day and for each of the probiotic groups, 100 ml MRS was inoculated with either the wild type or mutant probiotic strains and these cultures were incubated overnight at 37°C (5% CO_2_). Cells were harvested, washed twice in PBS and diluted in 1 ml PBS in order to obtain 1×10^11^ CFU/ml. 0.1 ml aliquots of the appropriately prepared cultures were administered to mice daily.

On the first and last day of the feeding trial (Day 0 and Day 7), fecal samples were collected in sterile containers. They were stored at 4°C until DNA extraction was performed (on the day of collection). All mice except 2 gave a fecal sample on the first day of the trial. The two mice belonged to the Bac− group and consequently pyrosequencing analysis was carried out on 3 samples instead of 5 in this group (Day 0, control group: *n* = 5, Bac+: *n* = 5 and Bac−: *n* = 3). At the end of the trial, we were able to collect samples from all mice (Day 7, control group: *n* = 5, Bac+: *n* = 5, Bac−: *n* = 5).

### Pig feeding trial

A total of 30 crossbred (Large White×Landrace) pigs (entire males) were weaned at *c*. 26 days of age. For the first 7 days post-weaning, pigs were fed an un-medicated starter diet (16.33 MJ digestible energy/kg and 15.5 g/kg lysine). This was followed by a 7-day acclimatization period, during which 100 ml of sterile reconstituted skim milk (RSM) was fed daily to each pig in addition to an un-medicated basal diet, which was formulated to contain 15.38 MJ digestible energy /kg and 14.7 g/kg total lysine. At 14 days post-weaning (Day 0), pigs were blocked by weight and ancestry. Within blocks, 24 pigs were randomly assigned to one of three treatment groups (control Bac+ or Bac− groups, each containing eight pigs) for the 29-day treatment period (day 0–29). Pigs were individually penned and each treatment group was housed in separate but identical rooms to prevent cross-contamination between groups. During the treatment period, in addition to the basal diet, each pig in the probiotic groups received 100 ml RSM containing 1×10^10^ CFU of *L. salivarius* UCC118 WT Rif (Bac+ group) or *L. salivarius* UCC118 Δ*abpt* Rif (Bac− group) (Table S1) daily, while pigs in the control group received 100 ml sterile RSM daily as well as basal diet as was done by Casey et al. and Gardiner et al. [8], [16]. At all times during the trial, pigs were provided with *ad libitum* access to fresh water and the basal diet. Administered cultures were prepared as follows: for each of the probiotic groups, 700 ml MRS broth was inoculated with one of the probiotic strain to be administered (1% inoculum) and incubated overnight at 37°C (5% CO_2_). Cells were harvested, washed twice in PBS and diluted in 7 litters of RSM in order to obtain 1×10^8^ CFU/ml. 100 ml aliquots of probiotic milk were stored at 4°C for no longer than 7 days before being administered to pigs.

On the first, 14^th^ and 28^th^ days of the feeding trial (Day 0, Day 14 and Day 28), following rectal stimulation, fecal samples were collected into sterile containers from each of the pigs. They were stored at 4°C until microbiological analysis was performed or −80°C for no more than two days until microbiota analysis was performed. Fecal samples were collected from all pigs but one on Day 0 and from all pigs on Day 28. A fecal sample was collected on Day 2, for the pig (Control group) that was not sampled on Day 0. On Day 29, all the pigs were slaughtered by captive bolt stunning followed by exsanguination and the entire GIT was removed. Immediately, 30 cm before the ileo–cecal junction, both ileal digesta and tissues were sampled for microbiological analysis. These samples were placed on ice for transport to the laboratory where they were stored at 4°C until microbiological analysis was performed (on the day of collection).

### Microbiological analysis of pig fecal and ileal samples

As *L. salivarius* is a natural inhabitant of the GIT, it is not possible here to differ between natural and administered *L. salivarius* and it is thus impossible to use molecular methods to assess the quantity of administered *L. salivarius* strains which have survive and/or colonize within the GIT. Thus, to allow enumeration of the probiotic strains used in this study, rifampicin-resistant variants of *L. salivarius* UCC118 WT and Δ*abpT*, named *L. salivarius* UCC118 WT Rif and UCC118 Δ*abpT* Rif (Table S1), were generated by selection on MRS agar plates supplemented with increasing concentration of rifampicin (5 to 150 µg/ml). Probiotic counts were obtained by homogenization of 1 g of fecal or ileal digesta samples in maximum recovery diluent (MRD; Bectin Dickinson, Franklin Lakes, NJ), followed by 10-fold dilutions in MRD and spread-plating on *Lactobacillus* selective agar (LBS; Becton Dickinson, Cockeysville, MD) supplemented with 150 µg/ml rifampicin as a selective agent and 50 U/ml nystatin (Sigma-Aldrich, St Louis, MO) to inhibit yeasts and moulds. Ileal tissue samples were rinsed gently in MRD to remove digesta and were further washed by immersing in MRD and shaking vigorously for 5 min. Tissue samples (1 g) were then homogenized in fresh MRD as 10-fold dilutions using a stomacher (Seward, London, UK). The resulting homogenate was further diluted 10-fold in MRD and appropriate dilutions were spread-plated on LBS supplemented with 150 µg/ml rifampin and 50 U/ml nystatin. These plates were incubated anaerobically at 37°C for 2 days.

### Determination of cytokine levels by ELISA experiments

Whole blood samples were taken from the anterior vena cava of each pig and collected in serum (Silicone-Coated Interior) collection tubes (BD Vacutainer Systems, Franklin Lakes, NJ) on days 0, 14 and 29. Samples were stored at room temperature for at least one hour. The serum fraction of each sample was then isolated by centrifugation and stored at −80°C until further use. Concentrations of IL-8 and IL-10 were determined in these fractions using porcine-specific cytokine ELISA kits (R&D Systems, Minneapolis, MN) in accordance with the manufacturer's instructions.

### Microbiota analysis

DNA was extracted from fecal samples using the QIAamp DNA Stool Mini Kit, according to standard protocol (Qiagen, West Sussex, UK). The V4 or V4–V5 regions of the 16S rRNA gene were amplified as described by Claesson and collaborators with small modifications in PCR conditions [38]. A single separate PCR was performed on each sample using universal 16S rRNA primers, listed in Table S2, and BIOTAQ DNA Polymerase, according to the manufacturer's recommendations (Bioline, London, United Kingdom). The PCR conditions were 94°C for 50 seconds (initialization and denaturing), 42°C for 30 seconds (annealing), 72°C for 60 seconds in 35 cycles (extension), and a final elongation step at 72°C for 5 minutes. Negative control reactions containing all components, but water instead of template, were performed to confirm lack of contamination with post-PCR product. PCR products were quantified using the Quant-iT™ PicoGreen® dsDNA Kit according to the manufacturer's conditions (Turner BioSystems, Inc., Sunnyvale, CA). Concentration of all obtained PCR products was around 50 ng/ml and they were thus considered homogeneous. Finally for each time point, 50 ng of amplicons corresponding to individual samples were pooled together.

Pooled 16S rRNA gene amplicons were then sequenced using 454 GS FLX Titanium technology (Cogenics, Meylan, France for the mouse trial and Macrogen, Geumchen-gu, South Korea for the pig trial). All sequence reads are deposited at the metagenomics analysis server MG-RAST (http://metagenomics.anl.gov/, Project ID 153).

For the mouse trial, pyrosequencing produced an average of 30,840 reads per subject and time point after quality filtering was applied using the RDP's Pyrosequencing Pipeline (at http://pyro.cme.msu.edu/init/form.spr) [39] of no ambiguous bases, exact primer, and 210 bp reads. Reads were clustered into operational taxonomic units (OTUs) at a 100% sequence identity threshold using the RDP's Pyrosequencing Pipeline [39].

For the pig trial, pyrosequencing produced an average of 29,830 reads per subject and time point after quality filtering was applied using the Qiime settings [40] of no ambiguous bases, a mean quality score above 25, a mean window quality score above 25, maximum homopolymer run not exceeding a limit of 6 and no mismatches in the primer, and trimming the error-prone 3′ ends at a length of 210 bp. Reads were clustered into operational taxonomic units (OTUs) at a 97% sequence identity threshold using Qiime [40].

Clustering analysis was then carried out using UniFrac distance [41] based PCoAs to provide a deep understanding of the structures within the datasets. Taxonomic level datasets were generated by mapping the reads to taxonomic levels using the RDP classifier [39]. A confidence value of 0.5 was considered a positive identification. Using these datasets, groups of samples could be compared at multiple phylogenetic depths. In order to control for varying number of reads between subjects, the overall data per subject was normalized by scaling to an intensity of 1.

To assign amplicon sequences to species level, we extracted 77,294 V4 sequences from 127,977 full-length 16S rRNA genes having complete species classifications (RDP release 10.24) using the same primer pair as was used for the amplification. In brief, an association table with species-specific cut-off BLAST scores was designed from an all-against-all BLAST search of the *in silico* extracted V4 sequences. If the same-species score for a certain species was higher than the score of the first hit against a different species, than that species was considered assignable, and the score of the first hit against a different species was recorded as the cut-off score. Thus, 53% of 9,664 species with extracted V4 sequences were assignable through this approach. Subsequently, all 9,645 and 271,059 OTUs in the porcine and murine data sets, respectively, were BLAST searched against this species database resulting in classification efficiencies of 2.6% and 5.3% for porcine and murine data sets, respectively, into species level (best BLAST hit against that species had to greater than its cut-off score).

### Statistical analysis

The processed Pyrosequencing datasets, microbial count dataset and the cytokine dataset were analyzed in R [42]. Feature selection was carried out using the Wilcoxon Rank-Sum test with correction for multiple testing using either Bonferroni correction or q-values where there were a large number of variables being tested. Q values were generated using the R library q value [43]. When applying statistics, rare taxa were removed using a filter of 20% occupancy. This means that when applying a statistical test to a dataset, a variable was removed from testing if it contained 80% or more zeros. For the mouse feeding trail the time-point 0 samples were grouped for the comparisons to the three treatment groups.

Statistical analysis of the pig growth performance was carried out as follows: three treatments were tested using twenty four entire male pigs arranged in eight randomized complete blocks. Each block consisted of three individual pigs similar in initial weight and ancestry. The experiment was analyzed using the General Linear Models (GLM) procedures of SAS (Sas Inst. Inc., Cary, NC) for a randomized complete block design. The pig was the experimental unit and the model used for the statistical analysis of pig performance had the effects for treatment. The results were presented as least squares means ± SEM. The Duncan's multiple range procedure was used for means separation.

Alpha diversity in murine and porcine microbiota datasets was calculated using both Shannon and Phylogenetic Diversity metrics [44]. The R statistical software was used to generate the statistics [42]. The Kruskal-Wallis test was used to determine statistical differences between the groups in the *Lactobacillus* genus as a whole and in *L. intestinalis*. The Mann-Whitney test was used to determine significant differences between each pair of groups.

## Supporting Information

Figure S1 **Construction and confirmation of the** ***abpT*** **gene deletion in** ***L. salivarius*** **UCC118.** A) Schematic representation of the Δ*abpT* mutant construction using the pORI_19_-pVE_6007_ system; see Materials and Methods for details. B) Confirmation of the *abpT* gene deletion by Southern hybridization. The 1 kb probe corresponds to the upper-region of the *abpT* gene. Expected lengths of genomic fragment recognized by the probe after digestion with *Eco*RV are shown. C) Bacteriocin production phenotype of UCC118 Bac+ and Bac− strains using an overlay method. Presence of a halo indicates sensitivity of the indicator strain (*L. sakei*) to the tested strain and thus indicates if bacteriocin is produced or not.
(PDF)

Figure S2 **Microbiota diversity analysis of mice and pigs administered with** ***L. salivarius*** **UCC118.** Panel A shows the mean value of two measures of alpha diversity for the six groups in the murine dataset, and Panel B shows the same parameters for the six groups in the porcine dataset.. The error bars are a measure of the standard error of the mean (S.E.M). The y-axis on the left indicates phylogenetic diversity and the y-axis on the right indicates the Shannon Index.
(PDF)

Table S1 **Bacterial strains and plasmids used in this study.**
(PDF)

Table S2 **Primers used in this study.**
(PDF)

Table S3 **Effect of** ***L. salivarius*** **UCC118 administration on the murine microbiota composition.**
(PDF)

Table S4 **Effect of** ***L. salivarius*** **UCC118 administration on the porcine microbiota composition.**
(PDF)
