# Supplementary material for: Effect of Lactobacillus salivarius Bacteriocin Abp118 on the Mouse and Pig Intestinal Microbiota
Source: PLoS One. 2012 Feb 17;7(2):e31113. doi: 10.1371/journal.pone.0031113 (PMC3281923; doi:10.1371/journal.pone.0031113)
Supplement: Figure S1 — Construction and confirmation of the abpT gene deletion in L. salivarius UCC118. A) Schematic representation of the ΔabpT mutant construction using the pORI19-pVE6007 system; see Materials and Methods for details. B) Confirmation of the abpT gene deletion by Southern hybridization. The 1 kb probe corresponds to the upper-region of the abpT gene. Expected lengths of genomic fragment recognized by the probe after digestion with EcoRV are shown. C) Bacteriocin production phenotype of UCC118 Bac+ and Bac− strains using an overlay method. Presence of a halo indicates sensitivity of the indicator strain (L. sakei) to the tested strain and thus indicates if bacteriocin is produced or not. (PDF) [file pone.0031113.s001.pdf]

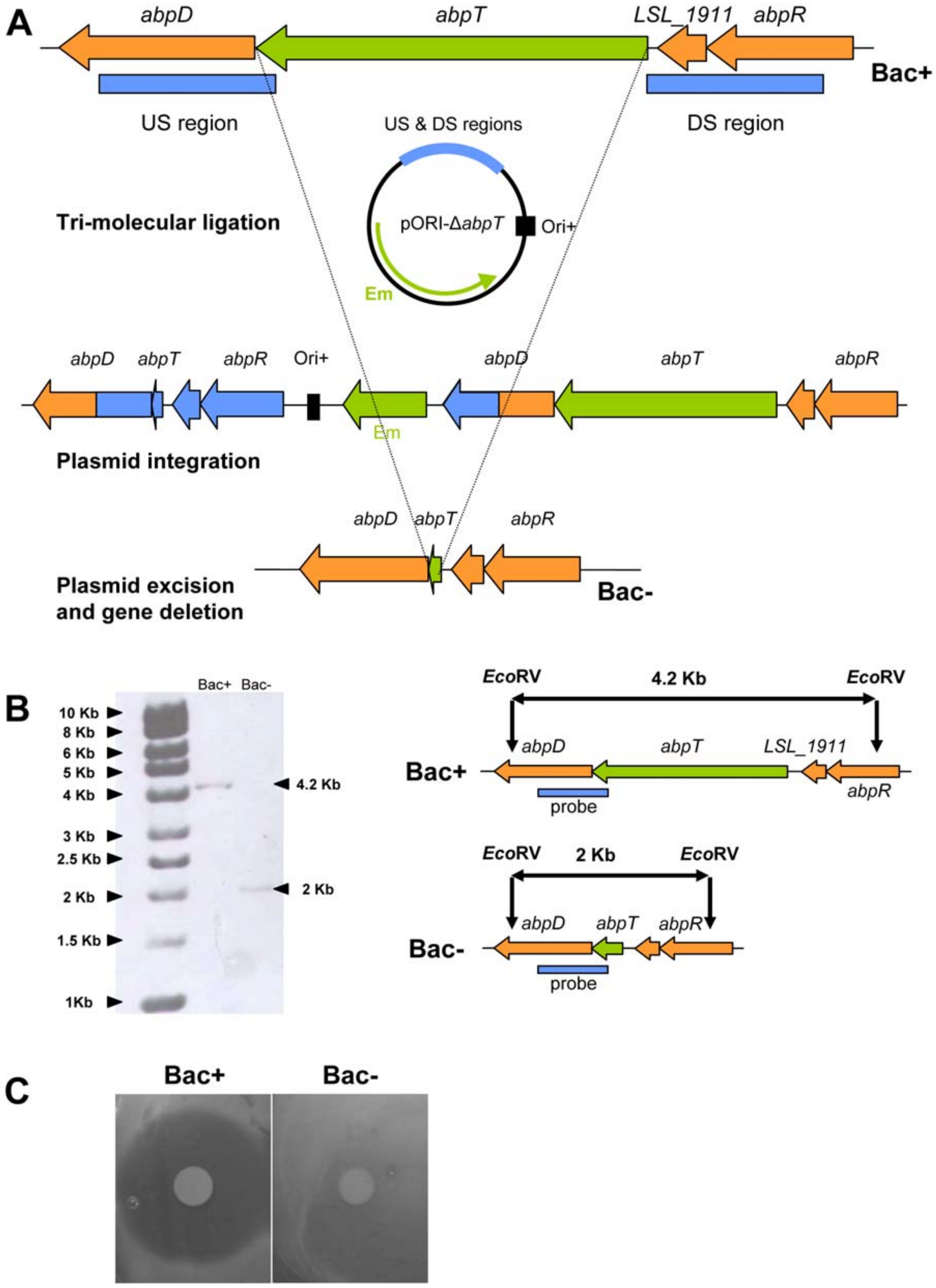

Figure S1. Construction and confirmation of the *abpT* gene deletion in *L. salivarius* UCC118. A) Schematic representation of the Δ*abpT* mutant construction using the pORI19-pVE6007 system; see Materials and Methods for details. B) Confirmation of the *abpT* gene deletion by Southern hybridization. The 1 kb probe corresponds to the upper-region of the *abpT* gene. Expected lengths of genomic fragment recognized by the probe after digestion with *Eco*RV are shown. C) Bacteriocin production phenotype of UCC118 Bac+ and Bac- strains using an overlay method. Presence of a halo indicates sensitivity of the indicator strain (*L. sakei*) to the tested strain and thus indicates if bacteriocin is produced or not.
