# Supplementary material for: Effect of Lactobacillus salivarius Bacteriocin Abp118 on the Mouse and Pig Intestinal Microbiota
Source: PLoS One. 2012 Feb 17;7(2):e31113. doi: 10.1371/journal.pone.0031113 (PMC3281923; doi:10.1371/journal.pone.0031113)
Supplement: Figure S2 — Microbiota diversity analysis of mice and pigs administered with L. salivarius UCC118. Panel A shows the mean value of two measures of alpha diversity for the six groups in the murine dataset, and Panel B shows the same parameters for the six groups in the porcine dataset.. The error bars are a measure of the standard error of the mean (S.E.M). The y-axis on the left indicates phylogenetic diversity and the y-axis on the right indicates the Shannon Index. (PDF) [file pone.0031113.s002.pdf]

A

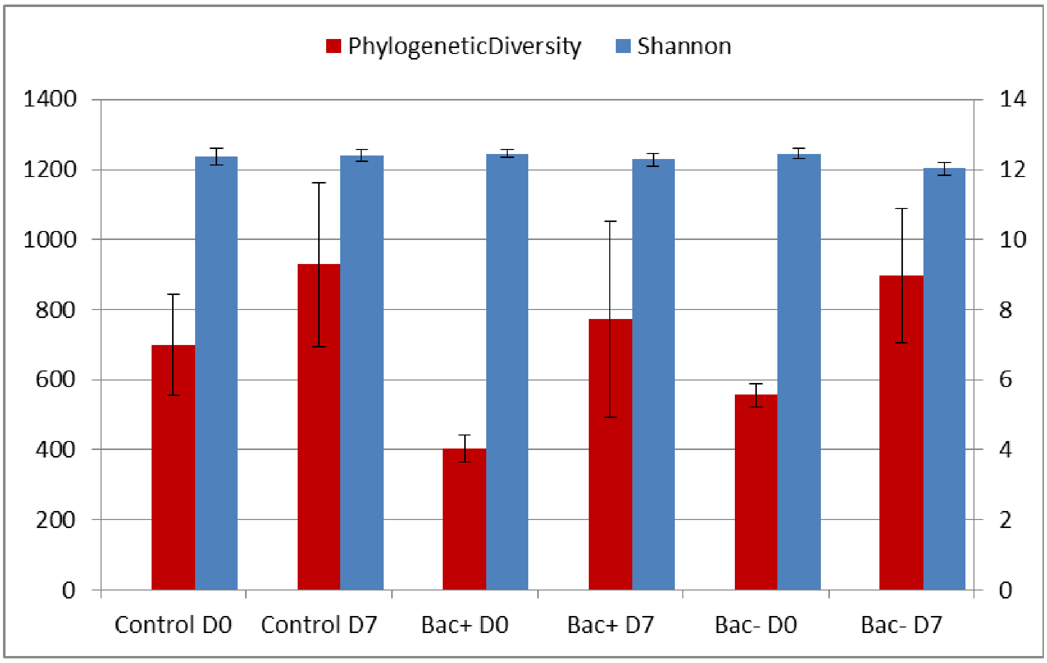

B

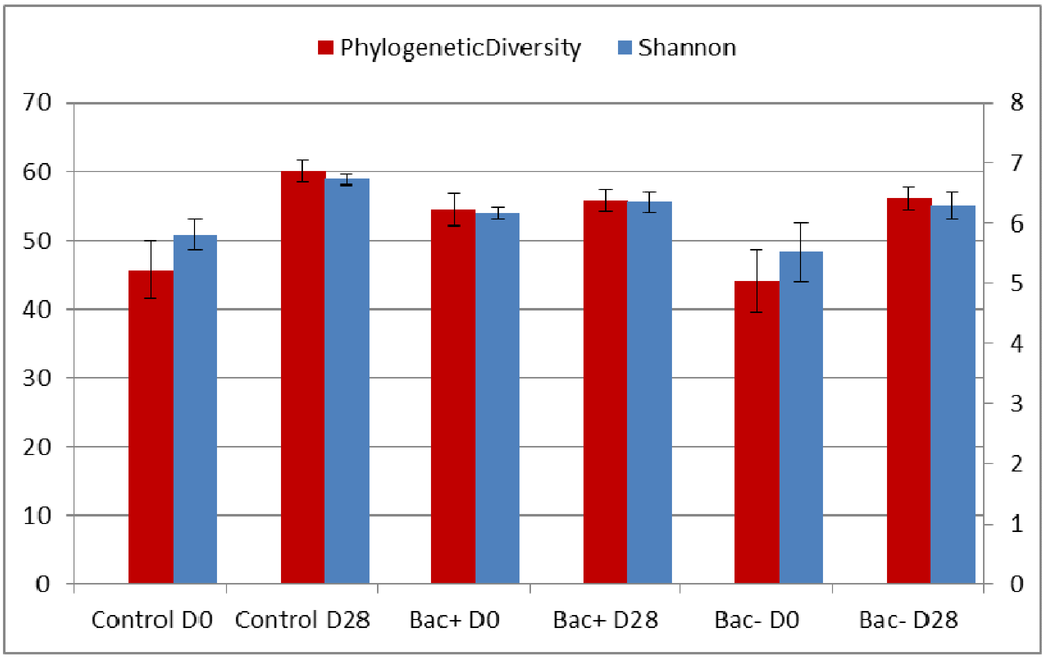

Figure S2. Microbiota diversity analysis of mice and pigs administered with *L. salivarius* UCC118. Panel A shows the mean value of two measures of alpha diversity for the six groups in the murine dataset, and Panel B shows the same parameters for the six groups in the porcine dataset. The error bars are a measure of the standard error of the mean (S.E.M). The y-axis on the left indicates Phylogenetic diversity and the y-axis on the right indicates the Shannon Index.
