# Supplementary material for: Effect of Lactobacillus salivarius Bacteriocin Abp118 on the Mouse and Pig Intestinal Microbiota
Source: PLoS One. 2012 Feb 17;7(2):e31113. doi: 10.1371/journal.pone.0031113 (PMC3281923; doi:10.1371/journal.pone.0031113)
Supplement: Table S1 — Bacterial strains and plasmids used in this study. (PDF) [file pone.0031113.s003.pdf]

Table S1: Bacterial strains and plasmids used in this study.

Bacterial strain or plasmid	Relevant characteristics	Reference or source
E. coli		
EC101	*repA*$^{+}$, pORI$_{19}$ host strain	[1]
L. lactis		
LL108	*repA*$^{+}$, pORI$_{19}$ host strain	[2]
L. salivarius		
UCC118 wild-type (WT)	Ileo-cecal isolate from a human adult	[3]
UCC118 pVE$_{6007}$	Strain harboring pVE$_{6007}$ plasmid (Cm^{R}, *repA*^{TS})	[4]
UCC118 *lacZ*	UCC118 *lacZ::pORI*$_{19}$, Em^{R}	[4]
UCC118 *abpT*	UCC118 *abpT::pORI*$_{19}$, Em^{R}, deficient in bacteriocin production	[5]
UCC118 Δ*abpT*	Clean deletion of the *abpT* gene, deficient in bacteriocin production	This study
UCC118 WT Rif	Rif^{R} derivative of UCC118	This study
UCC118 Δ*abpT* Rif	Rif^{R} derivative of UCC118 Δ*abpT*	This study
**Plasmids**		
pORI$_{19}$	Em^{R}, Ori^{+}, repA^{-}, *lacZ'*. Cloning vector	[1]
pORI-Δ*abpT*	pORI$_{19\text{-}1}$ containing flanking regions of *abpT*	This study

[1] Law J, Buist G, Haandrikman A, Kok J, Venema G, et al. (1995) A system to generate chromosomal mutations in *Lactococcus lactis* which allows fast analysis of targeted genes. J Bacteriol 177: 7011-7018.

[2] Leenhouts K, Bolhuis A, Venema G, Kok J (1998) Construction of a food-grade multiple-copy integration system for *Lactococcus lactis*. Appl Microbiol Biotechnol 49: 417-423.

[3] Dunne C, Murphy L, Flynn S, O'Mahony L, O'Halloran S, et al. (1999) Probiotics: from myth to reality. Demonstration of functionality in animal models of disease and in human clinical trials. Antonie Van Leeuwenhoek 76: 279-292.

[4] van Pijkeren JP, Canchaya C, Ryan KA, Li Y, Claesson MJ, et al. (2006) Comparative and functional analysis of sortase-dependent proteins in the predicted secretome of *Lactobacillus salivarius* UCC118. Appl Environ Microbiol 72: 4143-4153.

[5] Corr SC, Li Y, Riedel CU, O'Toole PW, Hill C, et al. (2007) Bacteriocin production as a mechanism for the antiinfective activity of *Lactobacillus salivarius* UCC118. Proc Natl Acad Sci U S A 104: 7617-7621.
