# Supplementary material for: Effect of Lactobacillus salivarius Bacteriocin Abp118 on the Mouse and Pig Intestinal Microbiota
Source: PLoS One. 2012 Feb 17;7(2):e31113. doi: 10.1371/journal.pone.0031113 (PMC3281923; doi:10.1371/journal.pone.0031113)
Supplement: Table S2 — Primers used in this study. (PDF) [file pone.0031113.s004.pdf]

Table S2: Primers used in this study.

Primer	Primer sequence[(a)](5'-3')	Use
abpT-US for	GATGGATCCGGTTTAGATACGTTTCTAGTTAC	cloning flanks of *abpT* in $pORI_{19-1}$
abpT-US rev	AGGGGGATTTTTCTTGTTAAGAAAGTATTTAGTTAATAGTTAGGGGGGAG	cloning flanks of *abpT* in $pORI_{19-1}$
abpT-DS for	AATACTCCCCCCTAACTATTAACTAAATACTTTCTTAACAAGAAAAATCC	cloning flanks of *abpT* in $pORI_{19-1}$
abpT-DS rev	CCGGAATTCGAGCAGATAAAGATGGCATAG	cloning flanks of *abpT* in $pORI_{19-1}$
abpT KO for	GGTTGGAACTTAGAGTATTCCAG	verifying *abpT* deletion
abpT KO rev	TCCTGCACCTTGGATAGTTATCTC	verifying *abpT* deletion
V4 for-1[(b)]	CGTATCGCCTCCCTCGCGCCATCAGACGAGTGCGTAYTGGGYDTAAAGNG[(d)]	amplifying V4 region of bacterial 16S rRNA gene
V4 rev[(b)]	GCCTTGCCAGCCCGCTCAGTACNVGGGTATCTAATCC[(d)]	amplifying V4 region of bacterial 16S rRNA gene
V4 for-2[(c)]	CCATCTCATCCCTGCGTGTCTCCGACTCAGACGAGTGCGTAYTGGGYDTAAAGNG[(d)]	amplifying V4-V5 region of bacterial 16S rRNA gene
V5 rev[(c)]	CCTATCCCCTGTGTGCCTTGGCAGTCTCAGCCGTCAATTYYTTTRAGTTT[(d)]	amplifying V4-V5 region of bacterial 16S rRNA gene

(a) From the annotated sequence of *L. salivarius* UCC118 [13].
(b) Primers used for the mouse trial pyrosequencing analysis.
(c) Primers used for the pig trial pyrosequencing analysis.
(d) Primers used for the pyrosequencing analysis. Each primer contains a sequence homologue to the V4 or V4-V5 region of the bacterial 16S rRNA gene and a 454 adaptor sequence (underlined) used for sequencing. Each forward primer contains in addition a barcode (in red) that was used to distinguish between each sample in the pool of amplicons (a total of 24 different barcodes were used in these studies).
