# Supplementary material for: Effect of Lactobacillus salivarius Bacteriocin Abp118 on the Mouse and Pig Intestinal Microbiota
Source: PLoS One. 2012 Feb 17;7(2):e31113. doi: 10.1371/journal.pone.0031113 (PMC3281923; doi:10.1371/journal.pone.0031113)
Supplement: Table S3 — Effect of L. salivarius UCC118 administration on the murine microbiota composition. (PDF) [file pone.0031113.s005.pdf]

Table S3: Effect of *L. salivarius* UCC118 administration on the murine microbiota composition.

	Control (a)		Bac+ (a)			Bac- (a)	
	Day 0	Day 7	Day 0	Day 7		Day 0	Day 7
**Phylum**							
Bacteroidetes	0.62500 (0.612 - 0.646)	0.56300 (0.554 - 0.635)	0.67800 (0.648 - 0.733)	0.56600 (0.433 - 0.66)		0.66000 (0.6 - 0.729)	0.68900 (0.625 - 0.736)
Firmicutes	0.33200 (0.318 - 0.335)	0.38100 (0.331 - 0.39)	0.30500 (0.248 - 0.305)	0.36900 (0.299 - 0.534)		0.28700 (0.237 - 0.348)	0.27700 (0.245 - 0.314)
Tenericutes	0.00508 (0.00105 - 0.0143)	0.00155 (0.00126 - 0.00775)	0.00711 (0.000467 - 0.0221)	0.00320 (0.00251 - 0.00385)		0.00054 (0.00034 - 0.00458)	0.00042 (3.7e-05 - 0.00162)
Proteobacteria	0.00382 (0.0029 - 0.00409)	0.00433 (0.00374 - 0.00459)	0.00277 (0.00273 - 0.00291)	0.00378 (0.00256 - 0.00392)		0.00503 (0.00344 - 0.00654)	0.00310 (0.0015 - 0.00542)
TM7	0.00120 (0.00081 - 0.00385)	0.00182 (0.000673 - 0.00203)	0.00102 (0.000762 - 0.00117)	0.00146 (0.00132 - 0.00173)		0.00145 (0.00131 - 0.00283)	0.00074 (0.000317 - 0.00316)
Cyanobacteria	0.00005 (0 - 8.22e-05)	0.00000 (0 - 0)	0.00000 (0 - 0)	0.00007 (4.21e-05 - 9.89e-05)		0.00003 (1.74e-05 - 0.000198)	0.00000 (0 - 7.39e-05)
Actinobacteria	0.00003 (3.25e-05 - 4e-05)	0.00004 (2.92e-05 - 5.14e-05)	0.00000 (0 - 0)	0.00010 (0 - 0.000675)		0.00003 (1.57e-05 - 0.000215)	0.00000 (0 - 0)
Verrucomicrobia	0.00000 (0 - 0)	0.00003 (0 - 2.75e-05)	0.00000 (0 - 3.33e-05)	0.00000 (0 - 0)		0.00000 (0 - 1.57e-05)	0.00000 (0 - 0)
Deferribacteres	0.00000 (0 - 0)	0.00000 (0 - 0)	0.00000 (0 - 0)	0.00000 (0 - 0)		0.00000 (0 - 0.000139)	0.00000 (0 - 0)
OD1	0.00000 (0 - 0)	0.00000 (0 - 0)	0.00000 (0 - 0)	0.00000 (0 - 0)		0.00000 (0 - 0)	0.00000 (0 - 0)
Euryarchaeota	0.00000 (0 - 0)	0.00000 (0 - 0)	0.00000 (0 - 0)	0.00000 (0 - 0)		0.00000 (0 - 1.74e-05)	0.00000 (0 - 0)
Gemmatimonadetes	0.00000 (0 - 0)	0.00000 (0 - 0)	0.00000 (0 - 0)	0.00000 (0 - 0)		0.00000 (0 - 1.74e-05)	0.00000 (0 - 0)
**Genus**							
Bacteroides	0.17400 (0.17 - 0.185)	0.10500 (0.0907 - 0.137)	0.09820 (0.0952 - 0.168)	0.03820 (0.0367 - 0.0517)	*	0.14600 (0.114 - 0.157)	0.08950 (0.0846 - 0.0945)
Alistipes	0.14800 (0.146 - 0.158)	0.17300 (0.155 - 0.175)	0.20100 (0.165 - 0.207)	0.20600 (0.139 - 0.222)		0.21100 (0.198 - 0.216)	0.21500 (0.192 - 0.245)
Parabacteroides	0.04200 (0.0358 - 0.0476)	0.04840 (0.0468 - 0.0524)	0.04540 (0.0387 - 0.0482)	0.04950 (0.0418 - 0.0516)		0.03190 (0.0309 - 0.0445)	0.04130 (0.0347 - 0.0507)
Lachnospiraceae Incertae Sedis (IS)	0.03800 (0.0281 - 0.043)	0.05580 (0.0527 - 0.0565)	0.03710 (0.0316 - 0.0592)	0.05440 (0.0489 - 0.0836)		0.04470 (0.0361 - 0.0507)	0.06340 (0.029 - 0.0761)
Lactobacillus	0.02670 (0.00716 - 0.0281)	0.02110 (0.00734 - 0.0215)	0.01690 (0.0143 - 0.0393)	0.04580 (0.0275 - 0.057)		0.01910 (0.0152 - 0.0198)	0.01310 (0.0099 - 0.0163)
Tannerella	0.01950 (0.0125 - 0.0236)	0.02890 (0.0242 - 0.0301)	0.02030 (0.0132 - 0.0273)	0.00829 (0.00725 - 0.00871)	*	0.02690 (0.0226 - 0.029)	0.03320 (0.0197 - 0.0418)
Dorea	0.00695 (0.00555 - 0.00895)	0.00697 (0.00659 - 0.0142)	0.00710 (0.00374 - 0.0111)	0.00790 (0.00736 - 0.0166)		0.00344 (0.00311 - 0.00485)	0.00697 (0.00441 - 0.00783)
Alkaliphilus	0.00631 (0.00442 - 0.00715)	0.00396 (0.00276 - 0.00786)	0.00352 (0.0028 - 0.00567)	0.00405 (0.0022 - 0.00445)		0.00297 (0.00207 - 0.00357)	0.00317 (0.00166 - 0.00321)
Ruminococcus	0.00537 (0.00432 - 0.0144)	0.00529 (0.00388 - 0.00647)	0.00200 (0.00183 - 0.00336)	0.00856 (0.00178 - 0.00871)		0.00446 (0.00402 - 0.00465)	0.00130 (0.000958 - 0.0024)
Anaeroplasma	0.00508 (0.00105 - 0.0143)	0.00155 (0.00126 - 0.00775)	0.00711 (0.000467 - 0.0221)	0.00320 (0.00247 - 0.00385)		0.00054 (0.00034 - 0.00457)	0.00042 (3.7e-05 - 0.00162)
Ruminococcaceae I.S	0.00306 (0.003 - 0.00544)	0.00457 (0.00322 - 0.00491)	0.00376 (0.0033 - 0.00603)	0.00612 (0.00561 - 0.00919)		0.00447 (0.0041 - 0.00582)	0.00613 (0.00328 - 0.00684)
Parasporobacterium	0.00251 (0.0022 - 0.00257)	0.00265 (0.0018 - 0.00431)	0.00170 (0.000861 - 0.0017)	0.00818 (0.00683 - 0.00977)	*	0.00252 (0.00178 - 0.00333)	0.00125 (0.000416 - 0.00281)
Johnsonella	0.00202 (0.00156 - 0.00231)	0.00265 (0.00205 - 0.00402)	0.00204 (0.00167 - 0.00242)	0.00395 (0.00334 - 0.00418)		0.00217 (0.00206 - 0.0037)	0.00317 (0.00296 - 0.00333)
Roseburia	0.00189 (0.00186 - 0.00472)	0.00168 (0.00152 - 0.00179)	0.00221 (0.000969 - 0.00333)	0.00222 (0.00198 - 0.00225)		0.00244 (0.00189 - 0.00288)	0.00177 (0.00132 - 0.002)
Faecalibacterium	0.00156 (0.00112 - 0.00176)	0.00214 (0.00188 - 0.00349)	0.00084 (0.000617 - 0.00103)	0.00438 (0.00338 - 0.00803)	*	0.00170 (0.00137 - 0.00266)	0.00240 (0.00192 - 0.00455)
Papillibacter	0.00156 (0.00152 - 0.00177)	0.00263 (0.00252 - 0.00266)	0.00197 (0.00174 - 0.00207)	0.00458 (0.00346 - 0.00469)	*	0.00145 (0.00101 - 0.00191)	0.00233 (0.00183 - 0.00273)
TM7_genera_I.S	0.00120 (0.00081 - 0.00385)	0.00182 (0.000673 - 0.00203)	0.00102 (0.000762 - 0.00117)	0.00146 (0.00132 - 0.00173)		0.00145 (0.00131 - 0.00283)	0.00074 (0.000317 - 0.00316)
Lachnospira	0.00108 (0.000411 - 0.00287)	0.00099 (0.000643 - 0.00251)	0.00061 (0.000524 - 0.00153)	0.00173 (0.00129 - 0.00198)		0.00116 (0.00114 - 0.00121)	0.00052 (0.000221 - 0.000741)
Sporobacter	0.00101 (0.000999 - 0.00196)	0.00265 (0.00242 - 0.00321)	0.00210 (0.00181 - 0.0039)	0.00521 (0.00425 - 0.00522)		0.00202 (0.00161 - 0.00376)	0.00405 (0.00383 - 0.00491)
Bryantella	0.00096 (0.000637 - 0.00163)	0.00054 (0.000537 - 0.00079)	0.00040 (0.000262 - 0.000653)	0.00085 (0.00063 - 0.00112)		0.00094 (0.000738 - 0.0012)	0.00081 (0.000776 - 0.00103)
Acetanaerobacterium	0.00088 (0.000759 - 0.00105)	0.00170 (0.00157 - 0.00198)	0.00107 (0.000699 - 0.00116)	0.00169 (0.00142 - 0.00199)		0.00150 (0.00149 - 0.00167)	0.00137 (0.00122 - 0.00146)
Clostridium	0.00084 (0.00076 - 0.00113)	0.00019 (0.000173 - 0.000951)	0.00044 (3e-04 - 0.000969)	0.00110 (0.000398 - 0.00145)		0.00188 (0.00113 - 0.00201)	0.00004 (0 - 0.00033)
Rikenella	0.00078 (0.000651 - 0.000799)	0.00118 (0.000962 - 0.0013)	0.00120 (0.000754 - 0.00177)	0.00090 (0.000528 - 0.000995)		0.00084 (0.000834 - 0.00127)	0.00144 (0.00122 - 0.00188)
Syntrophococcus	0.00072 (0.000506 - 0.00156)	0.00035 (0.000192 - 0.000376)	0.00029 (0.000204 - 0.00032)	0.00023 (6.64e-05 - 0.000421)		0.00108 (0.000697 - 0.00115)	0.00106 (0.000626 - 0.00177)
Weissella	0.00055 (0.00036 - 0.00076)	0.00041 (0.000153 - 0.000488)	0.00052 (0.000467 - 0.000543)	0.00040 (0.000263 - 0.00059)		0.00118 (0.001 - 0.00195)	0.00041 (0.000295 - 0.000416)
Turicibacter	0.00051 (0.000163 - 0.000959)	0.00039 (0.000173 - 0.00113)	0.00070 (0.000267 - 0.00104)	0.00171 (0.000664 - 0.00323)		0.00082 (0.000687 - 0.00157)	0.00004 (3.53e-05 - 0.000111)
Anaerotruncus	0.00048 (0.000432 - 0.000742)	0.00052 (0.000383 - 0.00076)	0.00040 (0.000251 - 0.00102)	0.00153 (0.000693 - 0.00388)		0.00058 (0.000415 - 0.0041)	0.00042 (0.000375 - 0.00123)
Leuconostoc	0.00042 (7.99e-05 - 0.000452)	0.00012 (7.67e-05 - 0.000206)	0.00014 (0.000116 - 0.000238)	0.00008 (6.64e-05 - 0.000113)		0.00028 (0.000246 - 0.000467)	0.00007 (3.53e-05 - 0.00011)

Erysipelothrix	0.00033 (0.00026 - 0.000405)	0.00046 (0.000173 - 0.000687)	0.00015 (3.59e-05 - 0.00017)	0.00113 (0.00063 - 0.00202)		0.00003 (1.74e-05 - 0.000411)	0.00018 (0 - 0.000333)
Croceibacter	0.00031 (0.000135 - 0.000325)	0.00004 (2.92e-05 - 5.5e-05)	0.00015 (7.18e-05 - 0.000436)	0.00004 (0 - 6.64e-05)		0.00000 (0 - 7.86e-05)	0.00011 (0.000106 - 0.000125)
Anaerovorax	0.00027 (7.99e-05 - 0.000329)	0.00023 (3.83e-05 - 0.000386)	0.00010 (7.26e-05 - 0.000167)	0.00011 (4.21e-05 - 0.000232)		0.00022 (0.000156 - 0.00023)	0.00011 (0 - 0.000148)
Guggenheimella	0.00020 (0.000185 - 0.00044)	0.00015 (0.000117 - 0.000347)	0.00024 (0.000146 - 0.000254)	0.00013 (9.95e-05 - 0.00121)		0.00029 (0.000267 - 0.000318)	0.00015 (3.68e-05 - 0.000212)
Hespellia	0.00014 (2.06e-05 - 0.00016)	0.00003 (0 - 0.000797)	0.00048 (0.000323 - 0.000533)	0.00030 (7.51e-05 - 0.000299)		0.00007 (5.05e-05 - 0.000107)	0.00004 (3.68e-05 - 0.000167)
Acetitomaculum	0.00014 (0.00012 - 0.000247)	0.00003 (2.57e-05 - 0.000385)	0.00007 (3.63e-05 - 6.79e-05)	0.00011 (6.64e-05 - 0.000178)		0.00003 (1.57e-05 - 0.000106)	0.00017 (0.000147 - 0.000212)
Lactococcus	0.00013 (4e-05 - 0.000181)	0.00006 (0 - 0.000205)	0.00009 (7.26e-05 - 0.000102)	0.00013 (0.000113 - 0.000249)		0.00025 (0.000248 - 0.000271)	0.00015 (3.53e-05 - 0.000185)
Enterococcus	0.00012 (8.22e-05 - 0.000181)	0.00008 (5.85e-05 - 0.00018)	0.00006 (3.59e-05 - 6.66e-05)	0.00013 (6.64e-05 - 0.00032)		0.00006 (3.15e-05 - 0.000339)	0.00004 (0 - 0.000141)
Peptostreptococcaceae I.S	0.00010 (3.62e-05 - 0.000236)	0.00008 (0 - 0.000308)	0.00000 (0 - 6.66e-05)	0.00000 (0 - 0.00033)		0.00000 (0 - 0.000109)	0.00000 (0 - 0)
Ethanoligenens	0.00010 (6.17e-05 - 0.000101)	0.00008 (5.78e-05 - 7.71e-05)	0.00010 (7.26e-05 - 0.000102)	0.00025 (0.000231 - 0.000488)	*	0.00000 (0 - 6.96e-05)	0.00007 (4.71e-05 - 7.39e-05)
Orientia	0.00010 (7.99e-05 - 0.000103)	0.00008 (5.5e-05 - 7.71e-05)	0.00004 (3.39e-05 - 8.73e-05)	0.00007 (3.55e-05 - 9.89e-05)		0.00011 (5.43e-05 - 0.000176)	0.00000 (0 - 3.68e-05)
Holdemania	0.00009 (7.99e-05 - 0.000226)	0.00012 (0.000116 - 0.00022)	0.00004 (3.39e-05 - 0.000116)	0.00004 (3.75e-05 - 0.000132)		0.00000 (0 - 1.81e-05)	0.00000 (0 - 4.71e-05)
Allobaculum	0.00007 (0 - 0.000452)	0.00044 (0.000422 - 0.000994)	0.00007 (0 - 0.000179)	0.00000 (0 - 3.3e-05)		0.00000 (0 - 0)	0.00000 (0 - 0)
Catenibacterium	0.00007 (2.06e-05 - 0.00016)	0.00003 (0 - 2.92e-05)	0.00000 (0 - 0)	0.00003 (0 - 3.32e-05)		0.00000 (0 - 0)	0.00000 (0 - 0)
Acinetobacter	0.00007 (0 - 0.000163)	0.00006 (3.83e-05 - 0.000103)	0.00000 (0 - 2e-04)	0.00004 (0 - 4.21e-05)		0.00007 (6.63e-05 - 0.000107)	0.00004 (0 - 3.7e-05)
Streptophyta	0.00005 (0 - 8.22e-05)	0.00000 (0 - 0)	0.00000 (0 - 0)	0.00007 (4.21e-05 - 9.89e-05)		0.00003 (1.74e-05 - 0.000198)	0.00000 (0 - 7.39e-05)
Fastidiosipila	0.00005 (4e-05 - 6.51e-05)	0.00000 (0 - 7.71e-05)	0.00003 (0 - 3.63e-05)	0.00004 (3.3e-05 - 8.43e-05)		0.00000 (0 - 5.43e-05)	0.00000 (0 - 0)
Prevotella	0.00004 (3.62e-05 - 6.17e-05)	0.00029 (0.000115 - 0.000308)	0.00000 (0 - 3.33e-05)	0.00729 (0.00375 - 0.0106)	*	0.00000 (0 - 3.15e-05)	0.00007 (0 - 0.000291)
Arcobacter	0.00004 (2.06e-05 - 6.51e-05)	0.00000 (0 - 0)	0.00000 (0 - 7.18e-05)	0.00003 (0 - 4.21e-05)		0.00013 (8.03e-05 - 0.000226)	0.00004 (3.53e-05 - 4.16e-05)
Coprococcus	0.00004 (0 - 5.43e-05)	0.00000 (0 - 0)	0.00000 (0 - 0)	0.00000 (0 - 0)		0.00000 (0 - 0)	0.00000 (0 - 0)
Shuttleworthia	0.00004 (0 - 6.51e-05)	0.00012 (2.92e-05 - 0.00023)	0.00000 (0 - 0)	0.00010 (0 - 0.000126)		0.00003 (1.57e-05 - 7e-05)	0.00005 (4.16e-05 - 0.000317)
Acetivibrio	0.00003 (0 - 6.51e-05)	0.00017 (0 - 0.000206)	0.00004 (0 - 5.82e-05)	0.00004 (0 - 0.000188)		0.00007 (3.62e-05 - 8.34e-05)	0.00000 (0 - 0)
Chryseobacterium	0.00003 (2.06e-05 - 3.62e-05)	0.00000 (0 - 0)	0.00000 (0 - 2.91e-05)	0.00000 (0 - 0)		0.00004 (3.38e-05 - 7.03e-05)	0.00000 (0 - 0)
Bifidobacterium	0.00003 (0 - 3.38e-05)	0.00003 (0 - 5.14e-05)	0.00000 (0 - 0)	0.00000 (0 - 0.000675)		0.00000 (0 - 0)	0.00000 (0 - 0)
Anaerostipes	0.00003 (2.06e-05 - 0.00012)	0.00003 (2.75e-05 - 8.77e-05)	0.00000 (0 - 3.63e-05)	0.00011 (0.000107 - 0.000126)		0.00000 (0 - 5.22e-05)	0.00005 (4.16e-05 - 7.05e-05)
Streptococcus	0.00003 (0 - 4.11e-05)	0.00000 (0 - 0)	0.00000 (0 - 2.91e-05)	0.00000 (0 - 3.3e-05)		0.00010 (9.94e-05 - 0.000233)	0.00000 (0 - 0)
Sporanaerobacter	0.00002 (0 - 4e-05)	0.00003 (2.89e-05 - 5.5e-05)	0.00013 (6.79e-05 - 0.000145)	0.00004 (3.55e-05 - 4.21e-05)		0.00007 (5.29e-05 - 0.000129)	0.00007 (3.68e-05 - 0.000125)
Shigella	0.00000 (0 - 0.000103)	0.00003 (0 - 7.67e-05)	0.00000 (0 - 0)	0.00004 (3.75e-05 - 9.95e-05)		0.00000 (0 - 0)	0.00000 (0 - 0)
Anaerofilum	0.00000 (0 - 2.06e-05)	0.00006 (2.57e-05 - 0.000192)	0.00004 (3.39e-05 - 7.18e-05)	0.00020 (3.32e-05 - 0.000263)		0.00007 (7.1e-05 - 9.91e-05)	0.00004 (0 - 0.000236)
Coprobacillus	0.00000 (0 - 7.99e-05)	0.00015 (5.85e-05 - 0.000308)	0.00003 (0 - 3.59e-05)	0.00010 (7.11e-05 - 0.000126)		0.00000 (0 - 1.57e-05)	0.00000 (0 - 0)
Akkermansia	0.00000 (0 - 0)	0.00003 (0 - 2.75e-05)	0.00000 (0 - 0)	0.00000 (0 - 0)		0.00000 (0 - 0)	0.00000 (0 - 0)
Desulfovibrio	0.00000 (0 - 0)	0.00003 (0 - 0.000137)	0.00003 (0 - 0.000116)	0.00007 (3.32e-05 - 0.000126)		0.00006 (3.15e-05 - 6.76e-05)	0.00009 (3.53e-05 - 0.000111)
Pontibacter	0.00000 (0 - 0)	0.00004 (2.92e-05 - 5.78e-05)	0.00000 (0 - 0)	0.00003 (0 - 3.55e-05)		0.00003 (3.31e-05 - 7.17e-05)	0.00007 (0 - 7.39e-05)
Citrobacter	0.00000 (0 - 3.62e-05)	0.00000 (0 - 0)	0.00000 (0 - 2.91e-05)	0.00003 (0 - 3.75e-05)		0.00006 (4.89e-05 - 6.76e-05)	0.00000 (0 - 0)
Sarcina	0.00000 (0 - 2.06e-05)	0.00000 (0 - 0)	0.00000 (0 - 0)	0.00003 (0 - 3.55e-05)		0.00004 (1.81e-05 - 4.95e-05)	0.00000 (0 - 0)
Mucispirillum	0.00000 (0 - 0)	0.00000 (0 - 0)	0.00000 (0 - 0)	0.00000 (0 - 0)		0.00000 (0 - 0.000139)	0.00000 (0 - 0)
Neoehrlichia	0.00000 (0 - 4e-05)	0.00003 (0 - 2.92e-05)	0.00000 (0 - 0)	0.00000 (0 - 0)		0.00003 (1.74e-05 - 3.55e-05)	0.00000 (0 - 0)
Thiobacter	0.00000 (0 - 3.62e-05)	0.00000 (0 - 5.14e-05)	0.00000 (0 - 2.91e-05)	0.00000 (0 - 3.75e-05)		0.00000 (0 - 0)	0.00000 (0 - 0)
Bilophila	0.00000 (0 - 0)	0.00000 (0 - 2.89e-05)	0.00000 (0 - 0)	0.00003 (0 - 4.21e-05)		0.00000 (0 - 1.74e-05)	0.00000 (0 - 0)
Eggerthella	0.00000 (0 - 0)	0.00000 (0 - 0)	0.00000 (0 - 0)	0.00000 (0 - 0)		0.00000 (0 - 0.000163)	0.00000 (0 - 0)
Abiotrophia	0.00000 (0 - 1.81e-05)	0.00000 (0 - 0)	0.00000 (0 - 0)	0.00000 (0 - 0)		0.00000 (0 - 1.81e-05)	0.00000 (0 - 0)
Anaerofustis	0.00000 (0 - 0)	0.00000 (0 - 0)	0.00000 (0 - 0)	0.00003 (0 - 3.55e-05)		0.00000 (0 - 0)	0.00000 (0 - 0)
Flavobacterium	0.00000 (0 - 0)	0.00000 (0 - 0)	0.00000 (0 - 0)	0.00000 (0 - 0)		0.00000 (0 - 1.81e-05)	0.00000 (0 - 0)
Erysipelotrichaceae I.S	0.00000 (0 - 0)	0.00000 (0 - 0)	0.00000 (0 - 0)	0.00000 (0 - 0)		0.00000 (0 - 0)	0.00000 (0 - 0)
Pediococcus	0.00000 (0 - 2.06e-05)	0.00000 (0 - 0)	0.00000 (0 - 0)	0.00000 (0 - 0)		0.00000 (0 - 0)	0.00000 (0 - 0)
Terrimonas	0.00000 (0 - 2.06e-05)	0.00000 (0 - 0)	0.00000 (0 - 0)	0.00000 (0 - 0)		0.00000 (0 - 0)	0.00000 (0 - 4.71e-05)
Microvirgula	0.00000 (0 - 2.06e-05)	0.00000 (0 - 0)	0.00000 (0 - 0)	0.00000 (0 - 0)		0.00000 (0 - 3.48e-05)	0.00000 (0 - 0)
Propionispira	0.00000 (0 - 0)	0.00000 (0 - 0)	0.00000 (0 - 0)	0.00000 (0 - 0)		0.00000 (0 - 0)	0.00000 (0 - 0)
Klebsiella	0.00000 (0 - 1.81e-05)	0.00000 (0 - 0)	0.00000 (0 - 0)	0.00000 (0 - 0)		0.00000 (0 - 1.57e-05)	0.00000 (0 - 0)
Veillonella	0.00000 (0 - 0)	0.00000 (0 - 0)	0.00000 (0 - 0)	0.00000 (0 - 0)		0.00003 (1.57e-05 - 3.38e-05)	0.00000 (0 - 0)
Enhydrobacter	0.00000 (0 - 0)	0.00000 (0 - 0)	0.00000 (0 - 0)	0.00000 (0 - 0)		0.00000 (0 - 0)	0.00000 (0 - 0)

Arcicella	0.00000 (0 - 0)	0.00000 (0 - 0)	0.00000 (0 - 0)	0.00000 (0 - 0)	0.00000 (0 - 3.48e-05)	0.00000 (0 - 0)
Verrucomicrobiaceae_genera_I.S	0.00000 (0 - 0)	0.00000 (0 - 0)	0.00000 (0 - 0)	0.00000 (0 - 0)	0.00000 (0 - 1.57e-05)	0.00000 (0 - 0)
Xylanibacter	0.00000 (0 - 0)	0.00000 (0 - 0)	0.00000 (0 - 0)	0.00000 (0 - 0)	0.00000 (0 - 0)	0.00000 (0 - 0)
Curtobacterium	0.00000 (0 - 0)	0.00000 (0 - 0)	0.00000 (0 - 0)	0.00000 (0 - 0)	0.00000 (0 - 1.81e-05)	0.00000 (0 - 0)
Pseudobutyrivibrio	0.00000 (0 - 0)	0.00000 (0 - 0)	0.00000 (0 - 0)	0.00000 (0 - 0)	0.00000 (0 - 0)	0.00000 (0 - 0)
Peptococcus	0.00000 (0 - 0)	0.00000 (0 - 0)	0.00000 (0 - 0)	0.00000 (0 - 3.32e-05)	0.00000 (0 - 0)	0.00000 (0 - 0)
Lachnobacterium	0.00000 (0 - 0)	0.00000 (0 - 0)	0.00000 (0 - 0)	0.00000 (0 - 0)	0.00000 (0 - 0)	0.00000 (0 - 0)
Bulleidia	0.00000 (0 - 0)	0.00000 (0 - 0)	0.00000 (0 - 0)	0.00000 (0 - 0)	0.00000 (0 - 0)	0.00000 (0 - 0)
Oribacterium	0.00000 (0 - 0)	0.00000 (0 - 0)	0.00000 (0 - 0)	0.00000 (0 - 0)	0.00000 (0 - 0)	0.00000 (0 - 0)
OD1_genera_I.S	0.00000 (0 - 0)	0.00000 (0 - 0)	0.00000 (0 - 0)	0.00000 (0 - 0)	0.00000 (0 - 0)	0.00000 (0 - 0)
Hallella	0.00000 (0 - 0)	0.00000 (0 - 0)	0.00000 (0 - 0)	0.00000 (0 - 0)	0.00000 (0 - 0)	0.00000 (0 - 0)
Subdoligranulum	0.00000 (0 - 0)	0.00000 (0 - 0)	0.00000 (0 - 0)	0.00000 (0 - 0)	0.00000 (0 - 0)	0.00000 (0 - 0)
Anaerosinus	0.00000 (0 - 0)	0.00000 (0 - 0)	0.00000 (0 - 0)	0.00000 (0 - 0)	0.00000 (0 - 1.74e-05)	0.00000 (0 - 0)
Pseudomonas	0.00000 (0 - 0)	0.00000 (0 - 0)	0.00000 (0 - 0)	0.00000 (0 - 0)	0.00000 (0 - 1.74e-05)	0.00000 (0 - 0)
Sutterella	0.00000 (0 - 0)	0.00000 (0 - 0)	0.00000 (0 - 0)	0.00000 (0 - 0)	0.00000 (0 - 0)	0.00000 (0 - 0)
Sulfurospirillum	0.00000 (0 - 0)	0.00000 (0 - 0)	0.00000 (0 - 0)	0.00000 (0 - 0)	0.00000 (0 - 0)	0.00000 (0 - 0)
Escherichia	0.00000 (0 - 0)	0.00000 (0 - 0)	0.00000 (0 - 0)	0.00000 (0 - 0)	0.00000 (0 - 0)	0.00000 (0 - 0)
Sporobacterium	0.00000 (0 - 0)	0.00000 (0 - 0)	0.00000 (0 - 0)	0.00000 (0 - 0)	0.00000 (0 - 0)	0.00000 (0 - 0)
Bacillus a	0.00000 (0 - 0)	0.00000 (0 - 0)	0.00000 (0 - 0)	0.00000 (0 - 0)	0.00000 (0 - 0)	0.00000 (0 - 0)
Verrucomicrobium	0.00000 (0 - 0)	0.00000 (0 - 0)	0.00000 (0 - 0)	0.00000 (0 - 0)	0.00000 (0 - 0)	0.00000 (0 - 0)
Sporacetigenium	0.00000 (0 - 0)	0.00000 (0 - 0)	0.00000 (0 - 0)	0.00000 (0 - 0)	0.00000 (0 - 0)	0.00000 (0 - 0)
Acidaminococcus	0.00000 (0 - 0)	0.00000 (0 - 0)	0.00000 (0 - 0)	0.00000 (0 - 0)	0.00000 (0 - 1.81e-05)	0.00000 (0 - 0)
Actinomyces	0.00000 (0 - 0)	0.00000 (0 - 0)	0.00000 (0 - 0)	0.00000 (0 - 0)	0.00000 (0 - 1.81e-05)	0.00000 (0 - 0)
Isobaculum	0.00000 (0 - 0)	0.00000 (0 - 0)	0.00000 (0 - 0)	0.00000 (0 - 0)	0.00000 (0 - 0)	0.00000 (0 - 0)
Gemmatimonas	0.00000 (0 - 0)	0.00000 (0 - 0)	0.00000 (0 - 0)	0.00000 (0 - 0)	0.00000 (0 - 1.74e-05)	0.00000 (0 - 0)
Stenotrophomonas	0.00000 (0 - 0)	0.00000 (0 - 0)	0.00000 (0 - 0)	0.00000 (0 - 0)	0.00000 (0 - 0)	0.00000 (0 - 0)
Aeromonas	0.00000 (0 - 0)	0.00000 (0 - 0)	0.00000 (0 - 0)	0.00000 (0 - 0)	0.00000 (0 - 1.57e-05)	0.00000 (0 - 0)
Niastella	0.00000 (0 - 0)	0.00000 (0 - 0)	0.00000 (0 - 0)	0.00000 (0 - 0)	0.00000 (0 - 0)	0.00000 (0 - 0)
Paralactobacillus	0.00000 (0 - 0)	0.00000 (0 - 0)	0.00000 (0 - 0)	0.00000 (0 - 0)	0.00000 (0 - 0)	0.00000 (0 - 0)
Dialister	0.00000 (0 - 0)	0.00000 (0 - 0)	0.00000 (0 - 0)	0.00000 (0 - 0)	0.00000 (0 - 0)	0.00000 (0 - 0)
Paracoccus	0.00000 (0 - 0)	0.00000 (0 - 0)	0.00000 (0 - 0)	0.00000 (0 - 0)	0.00000 (0 - 0)	0.00000 (0 - 0)
Arthrobacter	0.00000 (0 - 0)	0.00000 (0 - 0)	0.00000 (0 - 0)	0.00000 (0 - 0)	0.00000 (0 - 0)	0.00000 (0 - 0)
Acidovorax	0.00000 (0 - 0)	0.00000 (0 - 0)	0.00000 (0 - 0)	0.00000 (0 - 0)	0.00000 (0 - 0)	0.00000 (0 - 0)
Kineosphaera	0.00000 (0 - 0)	0.00000 (0 - 0)	0.00000 (0 - 0)	0.00000 (0 - 0)	0.00000 (0 - 0)	0.00000 (0 - 0)
Butyrivibrio	0.00000 (0 - 0)	0.00000 (0 - 0)	0.00000 (0 - 0)	0.00000 (0 - 0)	0.00000 (0 - 0)	0.00000 (0 - 0)
Oxobacter	0.00000 (0 - 0)	0.00000 (0 - 0)	0.00000 (0 - 0)	0.00000 (0 - 0)	0.00000 (0 - 0)	0.00000 (0 - 0)
Rubrivivax	0.00000 (0 - 0)	0.00000 (0 - 0)	0.00000 (0 - 0)	0.00000 (0 - 0)	0.00000 (0 - 0)	0.00000 (0 - 0)

(a) Values are median proportions of classified reads and interquartile range (in brackets) for each phylum or genus as determined by the RDP classifier and for each three groups (Control, Bac+, Bac-), n = 3-5.

*p < 0.005 and q < 0.05 between Bac+ group and control, on Day 7.
