# Supplementary material for: Effect of Lactobacillus salivarius Bacteriocin Abp118 on the Mouse and Pig Intestinal Microbiota
Source: PLoS One. 2012 Feb 17;7(2):e31113. doi: 10.1371/journal.pone.0031113 (PMC3281923; doi:10.1371/journal.pone.0031113)
Supplement: Table S4 — Effect of L. salivarius UCC118 administration on the porcine microbiota composition. (PDF) [file pone.0031113.s006.pdf]

Table S4: Effect of *L. salivarius* UCC118 administration on the porcine microbiota composition.

	Control (a)		Bac+ (a)			Bac- (a)	
	Day 0	Day 28	Day 0	Day 28		Day 0	Day 28
**Phylum**							
Firmicutes	0.49800 (0.414 - 0.536)	0.45100 (0.419 - 0.485)	0.50200 (0.456 - 0.513)	0.44900 (0.414 - 0.479)		0.52300 (0.506 - 0.545)	0.47500 (0.414 - 0.536)
Bacteroidetes	0.35700 (0.333 - 0.38)	0.28000 (0.255 - 0.33)	0.31100 (0.246 - 0.385)	0.34900 (0.26 - 0.39)		0.36000 (0.329 - 0.408)	0.33000 (0.285 - 0.355)
Spirochaetes	0.02040 (0.0161 - 0.0285)	0.15400 (0.133 - 0.169)	0.06230 (0.0129 - 0.107)	0.05150 (0.0382 - 0.0741)	*	0.01340 (0.00435 - 0.0217)	0.05020 (0.0394 - 0.0864)
Proteobacteria	0.00527 (0.00519 - 0.145)	0.01550 (0.00829 - 0.0211)	0.03410 (0.00867 - 0.0631)	0.03560 (0.0278 - 0.0786)		0.01230 (0.00765 - 0.0187)	0.04300 (0.0238 - 0.0571)
Tenericutes	0.00167 (0.0016 - 0.00519)	0.00118 (0.000907 - 0.00155)	0.00058 (0.000342 - 0.00399)	0.00501 (0.00154 - 0.00758)		0.00181 (0.00104 - 0.00195)	0.00349 (0.00211 - 0.00769)
Fibrobacteres	0.00079 (8.55e-05 - 0.00152)	0.00238 (0.000715 - 0.00433)	0.00001 (0 - 8.22e-05)	0.00086 (0.00064 - 0.00162)		0.00000 (0 - 0)	0.00004 (1.96e-05 - 0.00262)
Euryarchaeota	0.00009 (5.41e-05 - 9.67e-05)	0.00005 (0 - 6.72e-05)	0.00006 (4.86e-05 - 0.000239)	0.00010 (5.79e-05 - 0.000293)		0.00007 (4.92e-05 - 0.000134)	0.00013 (8.01e-05 - 0.000288)
TM7	0.00009 (7.99e-05 - 0.000114)	0.00005 (0 - 0.000125)	0.00000 (0 - 2.95e-05)	0.00000 (0 - 9.81e-06)		0.00000 (0 - 3.68e-05)	0.00000 (0 - 0)
Synergistetes	0.00009 (0 - 0.000176)	0.00004 (0 - 0.000113)	0.00008 (0 - 0.000172)	0.00016 (0.000119 - 0.000206)		0.00004 (0 - 0.000141)	0.00004 (3.74e-05 - 0.000418)
Actinobacteria	0.00008 (0 - 8.55e-05)	0.00017 (2.41e-05 - 0.00025)	0.00031 (0.000132 - 0.00892)	0.00022 (8.07e-05 - 0.000435)		0.00027 (6.16e-05 - 0.000449)	0.00004 (0 - 0.000171)
Fusobacteria	0.00006 (5.41e-05 - 0.000527)	0.00000 (0 - 0)	0.00007 (2.05e-05 - 0.000472)	0.00000 (0 - 0)		0.00009 (1.58e-05 - 0.000234)	0.00000 (0 - 0)
Deferribacteres	0.00005 (0 - 8.11e-05)	0.00063 (0.00028 - 0.00148)	0.00020 (0 - 0.000498)	0.00012 (4.51e-05 - 0.000586)		0.00000 (0 - 0.00038)	0.00050 (0.000206 - 0.00141)
Verrucomicrobia	0.00000 (0 - 0)	0.00026 (0.000156 - 0.000607)	0.00000 (0 - 2.19e-05)	0.00018 (0.000125 - 0.000301)		0.00002 (0 - 8.34e-05)	0.00025 (2.1e-05 - 0.000475)
Lentisphaerae	0.00000 (0 - 2.66e-05)	0.00010 (0 - 0.000545)	0.00007 (0 - 0.000317)	0.00006 (0 - 0.000119)		0.00000 (0 - 9.09e-05)	0.00017 (3.92e-05 - 0.000538)
Cyanobacteria	0.00000 (0 - 2.7e-05)	0.00000 (0 - 0)	0.00000 (0 - 0)	0.00000 (0 - 0)		0.00000 (0 - 0)	0.00000 (0 - 0)
Chlamydiae	0.00000 (0 - 0)	0.00000 (0 - 1.2e-05)	0.00000 (0 - 0)	0.00000 (0 - 0)		0.00000 (0 - 0)	0.00004 (0 - 6.01e-05)
Planctomycetes	0.00000 (0 - 0)	0.00000 (0 - 0)	0.00000 (0 - 0)	0.00000 (0 - 0)		0.00000 (0 - 0)	0.00000 (0 - 0)
**Genus**							
Prevotella	0.23500 (0.202 - 0.238)	0.09490 (0.0418 - 0.115)	0.18800 (0.153 - 0.26)	0.14700 (0.0738 - 0.21)		0.24700 (0.172 - 0.287)	0.08600 (0.0556 - 0.123)
Faecalibacterium	0.04270 (0.0413 - 0.0447)	0.02310 (0.0123 - 0.0289)	0.03570 (0.0283 - 0.0576)	0.01200 (0.00798 - 0.0183)		0.04400 (0.0345 - 0.0697)	0.01100 (0.00819 - 0.0261)
Blautia	0.02920 (0.0283 - 0.0525)	0.01560 (0.0119 - 0.0206)	0.02130 (0.0202 - 0.0414)	0.00618 (0.00495 - 0.01)		0.03540 (0.0187 - 0.0512)	0.01310 (0.00641 - 0.0141)
Butyricicoccus	0.02320 (0.0145 - 0.0236)	0.02040 (0.0147 - 0.0358)	0.02020 (0.0146 - 0.0267)	0.00650 (0.00564 - 0.018)		0.02110 (0.0127 - 0.0238)	0.01100 (0.00901 - 0.0152)
Coprococcus	0.02100 (0.0135 - 0.0243)	0.01120 (0.00745 - 0.0139)	0.01200 (0.00617 - 0.019)	0.00613 (0.00459 - 0.00994)		0.03220 (0.0185 - 0.0358)	0.00691 (0.00402 - 0.00943)
Oscillibacter	0.01670 (0.0164 - 0.0194)	0.02120 (0.014 - 0.0236)	0.02340 (0.0185 - 0.0264)	0.01000 (0.00907 - 0.011)		0.02370 (0.0213 - 0.03)	0.01260 (0.0117 - 0.0151)
Sporobacter	0.01450 (0.0104 - 0.0199)	0.02220 (0.018 - 0.0265)	0.01830 (0.0128 - 0.0244)	0.01430 (0.0117 - 0.019)		0.01880 (0.0131 - 0.0198)	0.01590 (0.0153 - 0.034)
Lactobacillus	0.01380 (0.0133 - 0.0138)	0.00416 (0.00272 - 0.0111)	0.00863 (0.00421 - 0.0155)	0.00997 (0.00683 - 0.0124)		0.01520 (0.0131 - 0.0278)	0.01190 (0.0099 - 0.0187)
Roseburia	0.01300 (0.00786 - 0.0408)	0.00206 (0.000632 - 0.00313)	0.00897 (0.00441 - 0.0203)	0.00716 (0.00243 - 0.0116)		0.01100 (0.00935 - 0.0127)	0.00368 (0.00255 - 0.0153)
Treponema	0.01180 (0.0096 - 0.0156)	0.14500 (0.0904 - 0.148)	0.03830 (0.0127 - 0.0987)	0.03750 (0.0271 - 0.0507)	*	0.01090 (0.00354 - 0.0179)	0.04670 (0.0341 - 0.0808)
Anaerophaga	0.01170 (0.00775 - 0.018)	0.00771 (0.00534 - 0.0111)	0.01160 (0.0028 - 0.032)	0.00481 (0.00264 - 0.00592)		0.00828 (0.00267 - 0.0137)	0.00375 (0.00195 - 0.00742)
Acidaminococcus	0.01150 (0.00768 - 0.0162)	0.00872 (0.00711 - 0.015)	0.01920 (0.00916 - 0.0344)	0.01150 (0.00841 - 0.0191)		0.01220 (0.0079 - 0.0178)	0.02640 (0.0105 - 0.0318)
Hydrogenoanaerobacterium	0.00782 (0.00768 - 0.0137)	0.00425 (0.00322 - 0.00687)	0.01140 (0.00857 - 0.0172)	0.00584 (0.00419 - 0.00767)		0.00373 (0.00131 - 0.00722)	0.01010 (0.01 - 0.0114)
Ruminococcus	0.00775 (0.00577 - 0.015)	0.00580 (0.0052 - 0.00825)	0.00665 (0.00497 - 0.00834)	0.01190 (0.00817 - 0.0126)		0.00879 (0.0077 - 0.0109)	0.01440 (0.00869 - 0.0265)
Parabacteroides	0.00571 (0.00462 - 0.00622)	0.00503 (0.00309 - 0.00993)	0.00158 (0.00108 - 0.00367)	0.00654 (0.00335 - 0.0153)		0.00571 (0.00398 - 0.00711)	0.00512 (0.00313 - 0.0103)
Dorea	0.00541 (0.00458 - 0.00687)	0.00193 (0.000567 - 0.00317)	0.00518 (0.00325 - 0.0105)	0.00105 (0.000577 - 0.00184)		0.00518 (0.00328 - 0.0063)	0.00075 (0.000564 - 0.00125)
Subdoligranulum	0.00392 (0.00382 - 0.00617)	0.00686 (0.00542 - 0.00806)	0.00653 (0.00377 - 0.00806)	0.01500 (0.00991 - 0.0184)	*	0.00525 (0.00491 - 0.00728)	0.01600 (0.00897 - 0.0207)
Anaerosporobacter	0.00249 (4e-04 - 0.0028)	0.00037 (0.00018 - 0.000847)	0.00064 (0.000505 - 0.00108)	0.00052 (0.000358 - 0.000591)		0.00100 (0.000567 - 0.0063)	0.00043 (0.000261 - 0.000882)
Papillibacter	0.00211 (0.00135 - 0.00237)	0.00215 (0.00151 - 0.00327)	0.00163 (0.00116 - 0.00217)	0.00230 (0.00101 - 0.0035)		0.00191 (0.000689 - 0.00406)	0.00314 (0.00178 - 0.00351)
Paraprevotella	0.00182 (0.00155 - 0.00239)	0.00053 (0.000228 - 0.000895)	0.00141 (0.00064 - 0.00201)	0.00072 (0.000281 - 0.00103)		0.00231 (0.0021 - 0.00316)	0.00093 (0.000361 - 0.0012)
Succinivibrio	0.00171 (0.000406 - 0.141)	0.00107 (0.000319 - 0.00868)	0.01950 (0.00451 - 0.0503)	0.01330 (0.0041 - 0.0679)		0.00748 (0.00243 - 0.0141)	0.02370 (0.00299 - 0.0319)
Mitsuokella	0.00170 (8.79e-05 - 0.00312)	0.00160 (0.000999 - 0.00272)	0.01180 (0.00405 - 0.0344)	0.00933 (0.00344 - 0.0224)		0.00250 (4.47e-05 - 0.008)	0.00691 (0.00382 - 0.0211)
Asteroleplasma	0.00167 (0.0016 - 0.00519)	0.00118 (0.000907 - 0.00155)	0.00058 (0.000342 - 0.00399)	0.00501 (0.00154 - 0.00758)		0.00181 (0.00104 - 0.00195)	0.00349 (0.00211 - 0.00769)

Anaerostipes	0.00141 (0.000712 - 0.00206)	0.00154 (0.00117 - 0.00211)	0.00048 (0.000354 - 0.00317)	0.00030 (0.000187 - 0.000765)	*	0.00068 (0.000508 - 0.00928)	0.00037 (8.11e-05 - 0.00119)
Bacteroides	0.00134 (0.00116 - 0.00387)	0.00365 (0.002 - 0.00541)	0.00212 (0.000841 - 0.00457)	0.00109 (0.000548 - 0.00229)		0.00232 (0.00117 - 0.00699)	0.00188 (0.000722 - 0.00667)
Lactonifactor	0.00128 (0.000967 - 0.00281)	0.00033 (0.000299 - 0.000758)	0.00076 (0.000428 - 0.00292)	0.00007 (4.15e-05 - 0.000169)	*	0.00192 (0.00143 - 0.00254)	0.00029 (0.000119 - 0.000378)
Sutterella	0.00114 (0.000568 - 0.00126)	0.00023 (0.000142 - 0.000283)	0.00047 (0.000442 - 0.00112)	0.00020 (0 - 0.000366)		0.00105 (0.000453 - 0.00238)	0.00012 (8.11e-05 - 0.000347)
Spirochaeta	0.00112 (0.000644 - 0.00384)	0.00237 (0.00212 - 0.00373)	0.00195 (0.000287 - 0.00357)	0.00241 (0.00104 - 0.00407)		0.00144 (0.000188 - 0.00419)	0.00192 (0.0015 - 0.0049)
Oribacterium	0.00111 (0.000612 - 0.00881)	0.00030 (0.000163 - 0.000414)	0.00312 (0.000611 - 0.00895)	0.00438 (0.00071 - 0.00841)	*	0.00061 (0.000468 - 0.000714)	0.00092 (0.000517 - 0.00404)
Parasporobacterium	0.00108 (0.00108 - 0.00114)	0.00014 (9.54e-05 - 0.000232)	0.00056 (0.00049 - 0.000933)	0.00014 (9.02e-05 - 0.000328)		0.00028 (7.16e-05 - 0.000602)	0.00024 (0 - 0.00042)
Butyricimonas	0.00096 (0.000622 - 0.00179)	0.00009 (2.9e-05 - 0.000169)	0.00137 (0.000762 - 0.00317)	0.00024 (4.22e-05 - 0.000635)		0.00070 (0.000651 - 0.000807)	0.00028 (0 - 0.000402)
Anaerotruncus	0.00085 (0.000805 - 0.000996)	0.00070 (0.000619 - 0.00102)	0.00066 (0.000444 - 0.000936)	0.00053 (0.00021 - 0.00111)		0.00079 (0.000582 - 0.00135)	0.00090 (0.000564 - 0.00102)
Fibrobacter	0.00079 (8.55e-05 - 0.00152)	0.00238 (0.000715 - 0.00433)	0.00001 (0 - 8.22e-05)	0.00086 (0.00064 - 0.00162)		0.00000 (0 - 0)	0.00004 (1.96e-05 - 0.00262)
Acetitomaculum	0.00059 (0.000487 - 0.000644)	0.00013 (7.22e-05 - 0.000411)	0.00038 (7.88e-05 - 0.000717)	0.00006 (2.94e-05 - 0.000112)		0.00009 (5.2e-05 - 0.000151)	0.00012 (4.18e-05 - 0.000374)
Catenibacterium	0.00057 (0.000381 - 0.00192)	0.00327 (0.00237 - 0.0208)	0.00294 (0.000331 - 0.00953)	0.01340 (0.00716 - 0.0207)		0.00032 (2.23e-05 - 0.000836)	0.00964 (0.00522 - 0.0165)
Peptococcus	0.00054 (0.000352 - 0.000772)	0.00066 (0.00044 - 0.000949)	0.00048 (0.000385 - 0.00101)	0.00042 (0.000297 - 0.000615)		0.00047 (0.000113 - 0.000939)	0.00038 (0.000225 - 0.000801)
Bulleidia	0.00054 (0.00029 - 0.000598)	0.00064 (0.000178 - 0.00143)	0.00047 (0.000227 - 0.000914)	0.00024 (9.2e-05 - 0.000718)		0.00030 (0.000142 - 0.000325)	0.00013 (9.5e-05 - 0.000207)
Acetivibrio	0.00053 (0.000439 - 0.00126)	0.00128 (0.000889 - 0.00156)	0.00057 (0.000431 - 0.00073)	0.00092 (0.000591 - 0.00102)		0.00081 (0.000281 - 0.00151)	0.00075 (0.000589 - 0.00141)
Pseudobutyrivibrio	0.00047 (0.000199 - 0.000906)	0.00008 (2.86e-05 - 0.000313)	0.00022 (3.7e-05 - 0.000526)	0.00000 (0 - 5.08e-05)		0.00017 (8.97e-05 - 0.000283)	0.00000 (0 - 0.000102)
Helicobacter	0.00042 (8.79e-05 - 0.000514)	0.00010 (2.86e-05 - 0.000278)	0.00003 (0 - 0.000271)	0.00012 (4.53e-05 - 0.000168)		0.00008 (7.91e-06 - 0.000123)	0.00008 (4.13e-05 - 0.000122)
Selenomonas	0.00041 (0.000107 - 0.00187)	0.00000 (0 - 0.000401)	0.00014 (0 - 0.00105)	0.00004 (2.96e-05 - 0.000112)		0.00000 (0 - 0)	0.00316 (0.000684 - 0.00485)
Mogibacterium	0.00040 (0.000243 - 0.000479)	0.00057 (0.000397 - 0.000709)	0.00046 (0.000218 - 0.000797)	0.00025 (0.000113 - 0.000497)		0.00041 (0.000212 - 0.000969)	0.00050 (0.000317 - 0.000606)
Acetanaerobacterium	0.00037 (0.000216 - 0.000387)	0.00040 (0.000258 - 0.00093)	0.00034 (0.000321 - 0.00043)	0.00016 (4.17e-05 - 0.000281)		0.00051 (0.000396 - 0.000797)	0.00029 (0.000137 - 0.00046)
Holdemania	0.00035 (0.000176 - 0.000399)	0.00014 (4.95e-05 - 0.000421)	0.00023 (0.000127 - 0.000326)	0.00019 (1e-04 - 0.000355)		0.00035 (0.000257 - 0.000619)	0.00008 (7.84e-05 - 0.000334)
Eubacterium	0.00032 (8.55e-05 - 0.0036)	0.00500 (0.00299 - 0.0067)	0.00051 (0.000304 - 0.00125)	0.00872 (0.00146 - 0.0129)		0.00039 (0.000102 - 0.000588)	0.00666 (0.00416 - 0.0136)
Anaerovibrio	0.00016 (6.44e-05 - 0.000703)	0.00018 (4.74e-05 - 0.000428)	0.00003 (2.05e-05 - 5.33e-05)	0.00016 (2.94e-05 - 0.000616)		0.00025 (0.000121 - 0.00037)	0.00007 (2.08e-05 - 0.000235)
Hallella	0.00010 (7.99e-05 - 0.000256)	0.00005 (3.86e-05 - 7.82e-05)	0.00019 (0.000111 - 0.000211)	0.00014 (0.000115 - 0.000261)	*	0.00005 (3.02e-05 - 8.88e-05)	0.00008 (0 - 0.000146)
Oxobacter	0.00009 (6.44e-05 - 0.000133)	0.00026 (0.000101 - 0.000399)	0.00018 (9.36e-05 - 0.000236)	0.00004 (0 - 0.00012)		0.00019 (0.000101 - 0.00025)	0.00004 (4e-05 - 0.000176)
TM7_genera_I.S	0.00009 (7.99e-05 - 0.000114)	0.00005 (0 - 0.000125)	0.00000 (0 - 2.95e-05)	0.00000 (0 - 9.81e-06)		0.00000 (0 - 3.68e-05)	0.00000 (0 - 0)
Robinsoniella	0.00009 (2.93e-05 - 9.67e-05)	0.00013 (0.000102 - 0.000289)	0.00004 (2.85e-05 - 0.000162)	0.00000 (0 - 5.01e-05)		0.00025 (1.51e-05 - 0.000822)	0.00008 (0 - 9.54e-05)
Methanosphaera	0.00009 (5.41e-05 - 8.79e-05)	0.00000 (0 - 3.83e-05)	0.00006 (2.69e-05 - 0.000127)	0.00006 (0 - 0.000178)		0.00002 (0 - 0.000113)	0.00008 (4e-05 - 0.000209)
Actinobacillus	0.00006 (2.93e-05 - 0.000186)	0.00000 (0 - 0)	0.00001 (0 - 2.64e-05)	0.00000 (0 - 0)		0.00002 (0 - 0.000107)	0.00000 (0 - 1.78e-05)
Fusobacterium	0.00006 (5.41e-05 - 0.000264)	0.00000 (0 - 0)	0.00006 (2.05e-05 - 0.000436)	0.00000 (0 - 0)		0.00009 (1.58e-05 - 0.000197)	0.00000 (0 - 0)
Campylobacter	0.00005 (5.33e-05 - 5.86e-05)	0.00007 (0 - 0.000127)	0.00007 (2.36e-05 - 0.000383)	0.00002 (0 - 9.29e-05)		0.00005 (1.24e-05 - 0.000104)	0.00008 (3.88e-05 - 8.11e-05)
Mucispirillum	0.00005 (0 - 8.11e-05)	0.00063 (0.00028 - 0.00148)	0.00020 (0 - 0.000498)	0.00012 (4.51e-05 - 0.000586)		0.00000 (0 - 0.00038)	0.00050 (0.000206 - 0.00141)
Megasphaera	0.00003 (0 - 0.00511)	0.00207 (0.000253 - 0.00356)	0.00571 (0.000118 - 0.0205)	0.00860 (0.00244 - 0.0128)		0.00100 (0.000153 - 0.00995)	0.00867 (0.0038 - 0.0143)
Barnesiella	0.00003 (0 - 5.7e-05)	0.00000 (0 - 5.58e-05)	0.00002 (0 - 5.88e-05)	0.00002 (0 - 9.3e-05)		0.00001 (0 - 2.64e-05)	0.00004 (1.96e-05 - 7.97e-05)
Clostridium	0.00003 (2.7e-05 - 0.000193)	0.01470 (0.00815 - 0.0332)	0.00018 (2.09e-05 - 0.00102)	0.00054 (0.000368 - 0.00162)		0.00018 (7.99e-05 - 0.000575)	0.00071 (8.12e-05 - 0.000973)
Ethanoligenens	0.00003 (0 - 5.33e-05)	0.00000 (0 - 3.66e-05)	0.00005 (2.42e-05 - 5.54e-05)	0.00000 (0 - 9.65e-06)		0.00003 (2.46e-05 - 3.12e-05)	0.00000 (0 - 1.96e-05)
Allisonella	0.00003 (2.66e-05 - 6.44e-05)	0.00000 (0 - 0)	0.00000 (0 - 0)	0.00000 (0 - 4.17e-05)		0.00000 (0 - 0)	0.00000 (0 - 4.18e-05)
Streptococcus	0.00003 (0 - 5.41e-05)	0.00022 (8.34e-05 - 0.00073)	0.00000 (0 - 3.45e-05)	0.00032 (4.09e-05 - 0.000855)		0.00003 (2.99e-05 - 4.47e-05)	0.00029 (0.000137 - 0.000587)
Alistipes	0.00003 (0 - 2.93e-05)	0.00008 (4.5e-05 - 0.000101)	0.00000 (0 - 6.19e-05)	0.00015 (7.21e-05 - 0.000269)		0.00001 (0 - 5.36e-05)	0.00021 (9.93e-05 - 0.000556)
Bifidobacterium	0.00000 (0 - 0)	0.00000 (0 - 0)	0.00009 (0 - 0.00818)	0.00000 (0 - 1.96e-05)		0.00001 (0 - 0.00014)	0.00000 (0 - 0)
Succiniclasticum	0.00000 (0 - 0)	0.00000 (0 - 2.52e-05)	0.00000 (0 - 0)	0.00002 (0 - 0.00563)		0.00000 (0 - 0)	0.00000 (0 - 0.00204)
Escherichia/Shigella	0.00000 (0 - 0)	0.00000 (0 - 9.53e-06)	0.00000 (0 - 1.36e-05)	0.00000 (0 - 4.17e-05)		0.00000 (0 - 9.49e-05)	0.00000 (0 - 3.74e-05)
Cetobacterium	0.00000 (0 - 0)	0.00000 (0 - 0)	0.00000 (0 - 7.41e-06)	0.00000 (0 - 0)		0.00000 (0 - 0)	0.00000 (0 - 0)
Desulfovibrio	0.00000 (0 - 2.66e-05)	0.00063 (0.000259 - 0.00119)	0.00003 (0 - 9.78e-05)	0.00093 (0.000396 - 0.00125)		0.00000 (0 - 2.23e-05)	0.00092 (0.000578 - 0.00192)
Akkermansia	0.00000 (0 - 0)	0.00000 (0 - 0)	0.00000 (0 - 0)	0.00000 (0 - 0)		0.00000 (0 - 0)	0.00000 (0 - 0)
Victivallis	0.00000 (0 - 2.66e-05)	0.00010 (0 - 0.000545)	0.00007 (0 - 0.000317)	0.00006 (0 - 0.000119)		0.00000 (0 - 9.09e-05)	0.00017 (3.92e-05 - 0.000538)
Orientia	0.00000 (0 - 0)	0.00026 (0.000177 - 0.000535)	0.00000 (0 - 6.61e-05)	0.00021 (6.35e-05 - 0.000488)		0.00000 (0 - 0)	0.00016 (8.01e-05 - 0.00046)
Veillonella	0.00000 (0 - 0)	0.00000 (0 - 0)	0.00000 (0 - 0)	0.00000 (0 - 0)		0.00000 (0 - 0)	0.00000 (0 - 0)
Paludibacter	0.00000 (0 - 2.7e-05)	0.00000 (0 - 0)	0.00000 (0 - 0)	0.00002 (0 - 5.03e-05)		0.00000 (0 - 0)	0.00000 (0 - 6.21e-05)
Pediococcus	0.00000 (0 - 0)	0.00000 (0 - 0)	0.00000 (0 - 0)	0.00000 (0 - 0)		0.00000 (0 - 0)	0.00000 (0 - 0)
Olsenella	0.00000 (0 - 0)	0.00002 (0 - 4.12e-05)	0.00005 (0 - 0.000305)	0.00011 (6.37e-05 - 2e-04)		0.00013 (3.7e-05 - 0.000207)	0.00000 (0 - 7.98e-05)

Galbibacter	0.00000 (0 - 0)	0.00007 (3.77e-05 - 0.000213)	0.00000 (0 - 5.03e-06)	0.00000 (0 - 1.93e-05)	0.00000 (0 - 0)	0.00000 (0 - 4.06e-05)
Subdivision5_genera_I.S	0.00000 (0 - 0)	0.00007 (0 - 0.000308)	0.00000 (0 - 0)	0.00004 (2.96e-05 - 0.000173)	0.00000 (0 - 3.68e-05)	0.00004 (0 - 2e-04)
Coprobacillus	0.00000 (0 - 2.66e-05)	0.00002 (0 - 0.00011)	0.00000 (0 - 3.45e-05)	0.00004 (2.89e-05 - 0.000155)	0.00000 (0 - 2.23e-05)	0.00000 (0 - 0.000178)
Marvinbryantia	0.00000 (0 - 2.66e-05)	0.00000 (0 - 0)	0.00007 (2.05e-05 - 0.000181)	0.00000 (0 - 5.3e-05)	0.00000 (0 - 9.09e-05)	0.00004 (0 - 6.25e-05)
Dialister	0.00000 (0 - 0)	0.00000 (0 - 0)	0.00000 (0 - 3.48e-05)	0.00000 (0 - 0)	0.00000 (0 - 0)	0.00000 (0 - 0)
Syntrophococcus	0.00000 (0 - 0)	0.00000 (0 - 4.08e-05)	0.00007 (2.84e-05 - 9.16e-05)	0.00004 (0 - 5.14e-05)	0.00001 (0 - 5.14e-05)	0.00004 (0 - 0.000137)
Sharpea	0.00000 (0 - 0)	0.00000 (0 - 0)	0.00000 (0 - 6.97e-06)	0.00006 (2.94e-05 - 0.000105)	0.00000 (0 - 0)	0.00004 (0 - 0.000165)
Cerasicoccus	0.00000 (0 - 0)	0.00000 (0 - 9.31e-05)	0.00000 (0 - 0)	0.00004 (2.96e-05 - 9.4e-05)	0.00000 (0 - 0)	0.00004 (0 - 0.000267)
Enterococcus	0.00000 (0 - 0)	0.00000 (0 - 0)	0.00000 (0 - 6.48e-06)	0.00000 (0 - 0)	0.00000 (0 - 0)	0.00000 (0 - 0)
Methanobrevibacter	0.00000 (0 - 0)	0.00002 (0 - 5.38e-05)	0.00000 (0 - 2.56e-05)	0.00006 (0 - 0.000148)	0.00002 (0 - 4.44e-05)	0.00004 (1.96e-05 - 0.000102)
Guggenheimella	0.00000 (0 - 0)	0.00000 (0 - 9.03e-06)	0.00000 (0 - 0)	0.00002 (0 - 0.000135)	0.00000 (0 - 0)	0.00000 (0 - 0.000102)
Leuconostoc	0.00000 (0 - 0)	0.00000 (0 - 3.31e-05)	0.00000 (0 - 3.91e-05)	0.00000 (0 - 0)	0.00001 (0 - 2.85e-05)	0.00004 (0 - 5.52e-05)
Propionispira	0.00000 (0 - 5.41e-05)	0.00000 (0 - 7.67e-05)	0.00000 (0 - 2.58e-05)	0.00000 (0 - 4.08e-05)	0.00000 (0 - 2.23e-05)	0.00000 (0 - 1.96e-05)
Croceibacter	0.00000 (0 - 0)	0.00002 (0 - 9.93e-05)	0.00000 (0 - 0)	0.00007 (2.94e-05 - 0.000129)	0.00000 (0 - 0)	0.00000 (0 - 6.29e-05)
Odoribacter	0.00000 (0 - 0)	0.00000 (0 - 0)	0.00000 (0 - 0)	0.00000 (0 - 1.06e-05)	0.00000 (0 - 2.23e-05)	0.00000 (0 - 0)
Lutispora	0.00000 (0 - 0)	0.00000 (0 - 3.66e-05)	0.00000 (0 - 8.19e-05)	0.00000 (0 - 0)	0.00000 (0 - 0)	0.00004 (0 - 3.92e-05)
Lactococcus	0.00000 (0 - 0)	0.00000 (0 - 0)	0.00000 (0 - 6.39e-06)	0.00000 (0 - 0)	0.00000 (0 - 0)	0.00000 (0 - 0)
Streptophyta	0.00000 (0 - 2.7e-05)	0.00000 (0 - 0)	0.00000 (0 - 0)	0.00000 (0 - 0)	0.00000 (0 - 0)	0.00000 (0 - 0)
Aeriscardovia	0.00000 (0 - 0)	0.00000 (0 - 0)	0.00000 (0 - 0)	0.00000 (0 - 0)	0.00000 (0 - 0)	0.00000 (0 - 0)
Howardella	0.00000 (0 - 2.85e-05)	0.00000 (0 - 0)	0.00000 (0 - 4.39e-05)	0.00000 (0 - 1.04e-05)	0.00000 (0 - 6.82e-05)	0.00000 (0 - 0)
Lachnobacterium	0.00000 (0 - 2.85e-05)	0.00000 (0 - 0)	0.00006 (0 - 7.53e-05)	0.00000 (0 - 0)	0.00000 (0 - 0)	0.00000 (0 - 1.96e-05)
Rothia	0.00000 (0 - 0)	0.00000 (0 - 3.6e-05)	0.00000 (0 - 0)	0.00000 (0 - 1.06e-05)	0.00000 (0 - 0)	0.00000 (0 - 0)
Turicibacter	0.00000 (0 - 0)	0.00000 (0 - 0.000189)	0.00000 (0 - 0)	0.00000 (0 - 0)	0.00000 (0 - 0)	0.00000 (0 - 0)
Tannerella	0.00000 (0 - 2.93e-05)	0.00000 (0 - 9.53e-06)	0.00000 (0 - 6.39e-06)	0.00000 (0 - 0)	0.00000 (0 - 1.84e-05)	0.00000 (0 - 3.92e-05)
Herbaspirillum	0.00000 (0 - 0)	0.00000 (0 - 1.6e-05)	0.00000 (0 - 0)	0.00000 (0 - 4.23e-05)	0.00000 (0 - 0)	0.00000 (0 - 6.16e-05)
Pyramidobacter	0.00000 (0 - 2.85e-05)	0.00000 (0 - 0)	0.00000 (0 - 0)	0.00000 (0 - 1.06e-05)	0.00000 (0 - 0)	0.00000 (0 - 4.13e-05)
Desulfomicrobium	0.00000 (0 - 0)	0.00000 (0 - 0)	0.00000 (0 - 0)	0.00000 (0 - 4.23e-05)	0.00000 (0 - 0)	0.00000 (0 - 0.000122)
Anaerofilum	0.00000 (0 - 8.11e-05)	0.00000 (0 - 0)	0.00000 (0 - 0)	0.00000 (0 - 0)	0.00000 (0 - 2.37e-05)	0.00000 (0 - 0)
Corynebacterium	0.00000 (0 - 0)	0.00000 (0 - 2.9e-05)	0.00000 (0 - 0)	0.00000 (0 - 9.85e-06)	0.00000 (0 - 0)	0.00000 (0 - 0)
Chlamydia	0.00000 (0 - 0)	0.00000 (0 - 0)	0.00000 (0 - 0)	0.00000 (0 - 0)	0.00000 (0 - 0)	0.00004 (0 - 6.01e-05)
Erysipelothrix	0.00000 (0 - 0)	0.00000 (0 - 1.91e-05)	0.00000 (0 - 0)	0.00000 (0 - 0)	0.00000 (0 - 0)	0.00000 (0 - 0)
Johnsonella	0.00000 (0 - 0)	0.00000 (0 - 0)	0.00000 (0 - 3.53e-05)	0.00000 (0 - 0)	0.00000 (0 - 0)	0.00000 (0 - 0)
Phascolarctobacterium	0.00000 (0 - 0)	0.00000 (0 - 0)	0.00000 (0 - 6.39e-06)	0.00000 (0 - 0)	0.00000 (0 - 1.84e-05)	0.00000 (0 - 0)
Schwartzia	0.00000 (0 - 0)	0.00000 (0 - 0)	0.00000 (0 - 0)	0.00000 (0 - 3.98e-05)	0.00000 (0 - 0)	0.00000 (0 - 0)
Acinetobacter	0.00000 (0 - 0)	0.00000 (0 - 0)	0.00000 (0 - 0)	0.00000 (0 - 0)	0.00000 (0 - 0)	0.00000 (0 - 0)
Oxalobacter	0.00000 (0 - 0)	0.00000 (0 - 9.03e-06)	0.00000 (0 - 0)	0.00000 (0 - 0)	0.00000 (0 - 0)	0.00000 (0 - 0)
Anaerofustis	0.00000 (0 - 0)	0.00000 (0 - 0)	0.00000 (0 - 0)	0.00000 (0 - 0)	0.00000 (0 - 0)	0.00000 (0 - 0)
Collinsella	0.00000 (0 - 0)	0.00000 (0 - 0)	0.00000 (0 - 2.56e-05)	0.00000 (0 - 0)	0.00000 (0 - 0)	0.00000 (0 - 0)
Parasutterella	0.00000 (0 - 0)	0.00000 (0 - 0)	0.00000 (0 - 0)	0.00000 (0 - 0)	0.00000 (0 - 0)	0.00000 (0 - 0)
Enterorhabdus	0.00000 (0 - 0)	0.00000 (0 - 0)	0.00000 (0 - 2.16e-05)	0.00000 (0 - 0)	0.00000 (0 - 0)	0.00000 (0 - 0)
Peptostreptococcus	0.00000 (0 - 0)	0.00000 (0 - 0)	0.00000 (0 - 6.82e-06)	0.00000 (0 - 0)	0.00000 (0 - 0)	0.00000 (0 - 0)
Anaerovorax	0.00000 (0 - 0)	0.00000 (0 - 9.53e-06)	0.00000 (0 - 0)	0.00000 (0 - 0)	0.00000 (0 - 0)	0.00000 (0 - 3.92e-05)
Heliobacillus	0.00000 (0 - 0)	0.00000 (0 - 8.02e-06)	0.00000 (0 - 0)	0.00000 (0 - 0)	0.00000 (0 - 0)	0.00000 (0 - 2.08e-05)
Gracilibacter	0.00000 (0 - 0)	0.00000 (0 - 0)	0.00000 (0 - 0)	0.00000 (0 - 0)	0.00000 (0 - 0)	0.00000 (0 - 0)
Citrobacter	0.00000 (0 - 0)	0.00000 (0 - 0)	0.00000 (0 - 0)	0.00000 (0 - 0)	0.00000 (0 - 0)	0.00000 (0 - 0)
Aerococcus	0.00000 (0 - 0)	0.00000 (0 - 0)	0.00000 (0 - 0)	0.00000 (0 - 0)	0.00000 (0 - 0)	0.00000 (0 - 0)
Blastopirellula	0.00000 (0 - 0)	0.00000 (0 - 0)	0.00000 (0 - 0)	0.00000 (0 - 0)	0.00000 (0 - 0)	0.00000 (0 - 0)
Thermogymnomonas	0.00000 (0 - 0)	0.00000 (0 - 0)	0.00000 (0 - 0)	0.00000 (0 - 0)	0.00000 (0 - 0)	0.00000 (0 - 0)
Pasteurella	0.00000 (0 - 0)	0.00000 (0 - 0)	0.00000 (0 - 0)	0.00000 (0 - 0)	0.00000 (0 - 0)	0.00000 (0 - 1.96e-05)
Anaerobiospirillum	0.00000 (0 - 0)	0.00000 (0 - 0)	0.00000 (0 - 0)	0.00000 (0 - 0)	0.00000 (0 - 0)	0.00000 (0 - 0)
Porphyromonas	0.00000 (0 - 0)	0.00000 (0 - 0)	0.00000 (0 - 0)	0.00000 (0 - 0)	0.00000 (0 - 0)	0.00000 (0 - 0)

Microvirgula	0.00000 (0 - 0)	0.00000 (0 - 0)	0.00000 (0 - 0)	0.00000 (0 - 0)	0.00000 (0 - 0)	0.00000 (0 - 0)
Weissella	0.00000 (0 - 0)	0.00000 (0 - 0)	0.00000 (0 - 0)	0.00000 (0 - 1.04e-05)	0.00000 (0 - 0)	0.00000 (0 - 0)
Allobaculum	0.00000 (0 - 0)	0.00000 (0 - 0)	0.00000 (0 - 0)	0.00000 (0 - 0)	0.00000 (0 - 0)	0.00000 (0 - 0)
Arthrobacter	0.00000 (0 - 0)	0.00000 (0 - 0)	0.00000 (0 - 0)	0.00000 (0 - 0)	0.00000 (0 - 0)	0.00000 (0 - 0)
Enhydrobacter	0.00000 (0 - 0)	0.00000 (0 - 0)	0.00000 (0 - 0)	0.00000 (0 - 0)	0.00000 (0 - 0)	0.00000 (0 - 0)
Xylanibacter	0.00000 (0 - 0)	0.00000 (0 - 0)	0.00000 (0 - 0)	0.00000 (0 - 0)	0.00000 (0 - 0)	0.00000 (0 - 0)
Moryella	0.00000 (0 - 0)	0.00000 (0 - 0)	0.00000 (0 - 0)	0.00000 (0 - 0)	0.00000 (0 - 0)	0.00000 (0 - 0)
Brucella	0.00000 (0 - 0)	0.00000 (0 - 0)	0.00000 (0 - 0)	0.00000 (0 - 0)	0.00000 (0 - 0)	0.00000 (0 - 0)
Natronincola	0.00000 (0 - 0)	0.00000 (0 - 0)	0.00000 (0 - 6.48e-06)	0.00000 (0 - 0)	0.00000 (0 - 0)	0.00000 (0 - 0)
Slackia	0.00000 (0 - 0)	0.00000 (0 - 0)	0.00000 (0 - 0)	0.00000 (0 - 0)	0.00000 (0 - 0)	0.00000 (0 - 0)
Peptoniphilus	0.00000 (0 - 0)	0.00000 (0 - 0)	0.00000 (0 - 0)	0.00000 (0 - 0)	0.00000 (0 - 0)	0.00000 (0 - 0)
Hespellia	0.00000 (0 - 0)	0.00000 (0 - 0)	0.00000 (0 - 0)	0.00000 (0 - 0)	0.00000 (0 - 0)	0.00000 (0 - 0)
Cloacibacillus	0.00000 (0 - 0)	0.00000 (0 - 0)	0.00000 (0 - 0)	0.00000 (0 - 9.65e-06)	0.00000 (0 - 0)	0.00000 (0 - 0)
Bosea	0.00000 (0 - 0)	0.00000 (0 - 0)	0.00000 (0 - 0)	0.00000 (0 - 0)	0.00000 (0 - 0)	0.00000 (0 - 0)
Comamonas	0.00000 (0 - 0)	0.00000 (0 - 0)	0.00000 (0 - 0)	0.00000 (0 - 0)	0.00000 (0 - 0)	0.00000 (0 - 0)
Hellea	0.00000 (0 - 0)	0.00000 (0 - 0)	0.00000 (0 - 0)	0.00000 (0 - 0)	0.00000 (0 - 0)	0.00000 (0 - 0)
Alcaligenes	0.00000 (0 - 0)	0.00000 (0 - 0)	0.00000 (0 - 0)	0.00000 (0 - 0)	0.00000 (0 - 0)	0.00000 (0 - 0)
Shuttleworthia	0.00000 (0 - 0)	0.00000 (0 - 0)	0.00000 (0 - 0)	0.00000 (0 - 0)	0.00000 (0 - 0)	0.00000 (0 - 0)
Klebsiella	0.00000 (0 - 0)	0.00000 (0 - 0)	0.00000 (0 - 0)	0.00000 (0 - 0)	0.00000 (0 - 0)	0.00000 (0 - 0)
Butyrivibrio	0.00000 (0 - 0)	0.00000 (0 - 0)	0.00000 (0 - 0)	0.00000 (0 - 0)	0.00000 (0 - 0)	0.00000 (0 - 0)
Acetobacter	0.00000 (0 - 0)	0.00000 (0 - 0)	0.00000 (0 - 0)	0.00000 (0 - 0)	0.00000 (0 - 0)	0.00000 (0 - 0)
Vitreoscilla	0.00000 (0 - 0)	0.00000 (0 - 0)	0.00000 (0 - 0)	0.00000 (0 - 0)	0.00000 (0 - 0)	0.00000 (0 - 0)
Actinomyces	0.00000 (0 - 0)	0.00000 (0 - 0)	0.00000 (0 - 0)	0.00000 (0 - 0)	0.00000 (0 - 0)	0.00000 (0 - 0)
Pigmentiphaga	0.00000 (0 - 0)	0.00000 (0 - 0)	0.00000 (0 - 0)	0.00000 (0 - 0)	0.00000 (0 - 0)	0.00000 (0 - 0)
Ornithobacterium	0.00000 (0 - 0)	0.00000 (0 - 0)	0.00000 (0 - 0)	0.00000 (0 - 0)	0.00000 (0 - 0)	0.00000 (0 - 0)
Haemophilus	0.00000 (0 - 0)	0.00000 (0 - 0)	0.00000 (0 - 0)	0.00000 (0 - 0)	0.00000 (0 - 0)	0.00000 (0 - 0)
Chryseobacterium	0.00000 (0 - 0)	0.00000 (0 - 0)	0.00000 (0 - 0)	0.00000 (0 - 0)	0.00000 (0 - 0)	0.00000 (0 - 0)
Lentisphaera	0.00000 (0 - 0)	0.00000 (0 - 0)	0.00000 (0 - 0)	0.00000 (0 - 0)	0.00000 (0 - 0)	0.00000 (0 - 0)
Flavobacterium	0.00000 (0 - 0)	0.00000 (0 - 0)	0.00000 (0 - 0)	0.00000 (0 - 0)	0.00000 (0 - 0)	0.00000 (0 - 0)
Sporobacterium	0.00000 (0 - 0)	0.00000 (0 - 0)	0.00000 (0 - 0)	0.00000 (0 - 0)	0.00000 (0 - 0)	0.00000 (0 - 0)
Psychrilyobacter	0.00000 (0 - 0)	0.00000 (0 - 0)	0.00000 (0 - 0)	0.00000 (0 - 0)	0.00000 (0 - 0)	0.00000 (0 - 0)
Anaerobacter	0.00000 (0 - 0)	0.00000 (0 - 0)	0.00000 (0 - 0)	0.00000 (0 - 0)	0.00000 (0 - 0)	0.00000 (0 - 0)
Pelagibacter	0.00000 (0 - 0)	0.00000 (0 - 0)	0.00000 (0 - 0)	0.00000 (0 - 0)	0.00000 (0 - 0)	0.00000 (0 - 0)
Filifactor	0.00000 (0 - 0)	0.00000 (0 - 0)	0.00000 (0 - 0)	0.00000 (0 - 0)	0.00000 (0 - 0)	0.00000 (0 - 0)
Asaccharobacter	0.00000 (0 - 0)	0.00000 (0 - 0)	0.00000 (0 - 0)	0.00000 (0 - 0)	0.00000 (0 - 0)	0.00000 (0 - 0)
Leifsonia	0.00000 (0 - 0)	0.00000 (0 - 0)	0.00000 (0 - 0)	0.00000 (0 - 0)	0.00000 (0 - 0)	0.00000 (0 - 0)
Gemella	0.00000 (0 - 0)	0.00000 (0 - 0)	0.00000 (0 - 0)	0.00000 (0 - 0)	0.00000 (0 - 0)	0.00000 (0 - 0)
Geosporobacter	0.00000 (0 - 0)	0.00000 (0 - 0)	0.00000 (0 - 0)	0.00000 (0 - 0)	0.00000 (0 - 0)	0.00000 (0 - 0)
Anaeroglobus	0.00000 (0 - 0)	0.00000 (0 - 0)	0.00000 (0 - 0)	0.00000 (0 - 0)	0.00000 (0 - 0)	0.00000 (0 - 0)

(a) Values are median proportions of classified reads and interquartile range (in brackets) for each phylum or genus as determined by the RDP classifier and for each three groups (Control, Bac+, Bac-), $n = 5\text{-}8$.

* $p < 0.01$ and Bonferroni Correction (phylum level) or q value (genus level) < 0.05 between Bac+ group and control, on Day 28.
